# “Science Manipulates the Things and Lives in Them”: Reconsidering Approach-Avoidance Operationalization Through a Grounded Cognition Perspective

**DOI:** 10.3389/fpsyg.2019.01418

**Published:** 2019-06-25

**Authors:** Ivane Nuel, Marie-Pierre Fayant, Theodore Alexopoulos

**Affiliations:** Laboratoire de Psychologie Sociale, Université de Paris, Boulogne-Billancourt, France

**Keywords:** approach-avoidance, grounded cognition, evaluations, construct validity, virtual reality

## Abstract

Approach and avoidance orientations are key elements of adaptive regulation at the evaluation-behavior interface. On the one hand, continuous evaluations of the world fuel approach-avoidance reactions as a function of the individual’s immediate environment. On the other hand, in turn these individual-environment adjustments influence evaluations. A grounded perspective of social cognition, placing the sensorimotor aspects of individual-environment interactions at the core of cognition, has much to offer for the understanding of evaluative processes. Despite the growing enthusiasm for a grounded view of cognition and action in the approach-avoidance literature, its core principles are seldom reflected at the operationalization level. In this paper, we relied on the insights of a grounded perspective to propose more encompassing operationalizations of approach-avoidance orientations and investigate their influence on evaluations. Across six studies, we varied the approach-avoidance operationalizations (upper-body incline, upper-body posture and walking steps) and incrementally considered the grounded assumptions. We failed to obtain the theorized positive effect of approach (as compared to avoidance) on evaluations. Interestingly, further exploratory analyses on two studies conducted in Virtual Reality suggested that the more participants felt being *present* in the situation, the more the approach-avoidance ecological actions activated the corresponding neuropsychological systems. We discuss these emergent findings in light of grounded cognition and the notion of feeling of presence.

## Introduction

“Science manipulates things and gives up living in them” Merleau-Ponty (1964). Individuals’ interactions with their social world are steered by two fundamental forces: approach and avoidance — i.e., the energization to move toward or away (Price and Harmon-Jones, 2016). The literature shows a flexible two-way influence between approach-avoidance and the way people evaluate their environment. Such an interplay enables individuals to tailor their behavior to the current challenges and constraints of the immediate situation. A grounded cognition perspective has much to say about approach-avoidance orientations, as it specifically addresses the dynamic interactions between the brain, the body, and the environment. However, we contend that up to now experimental implementations of approach-avoidance have not fully exploited the theoretical insights provided by a grounded view of cognition. In this paper, the major goal is to capitalize on the grounded cognition perspective, which offers a useful theoretical toolbox to conceive appropriate and warranted operationalizations of approach-avoidance orientations. In doing so, we aim to circumvent the limitations of previous research and to offer a more ecological investigation of the influence of approach and avoidance on social information processing.

Approach and avoidance represent the elemental energization and direction of behavior for a majority of living organisms (from unicellular ancestors to more complex ones). Humans, like every organism, are able to adapt to their dynamic environments by reducing the distance toward appetitive stimuli and increasing the distance vis-à-vis noxious stimuli in keeping with their survival (Schneirla, 1959; Elliot and Covington, 2001; McNaughton et al., 2016). Hence, individuals’ survival strongly depends on their ability to spontaneously detect approachable and/or avoidable entities (objects, people, events, ideas). This detection is assumed to spontaneously trigger appropriate behaviors (Chen and Bargh, 1999; Alexopoulos and Ric, 2007; Rougier et al., 2018). On a majority of cases, entities that entail a positive value for the organism trigger approach while those entailing a negative value trigger avoidance. Concerning interpersonal situations, research shows that, during social interactions, people tend to approach others if they seem trustworthy (Slepian et al., 2012), are smiling (Stins et al., 2011) or belong to the same group (Paladino and Castelli, 2008); but tend to avoid them if they display anger (Stins et al., 2011) or represent members of stereotyped and prejudiced groups (Word et al., 1974; Neumann et al., 2004; Paladino and Castelli, 2008). At the same time, when individuals are engaged in approach or avoidance behaviors, their cognitive activity is tuned to meet the specific requirements for goal attainment. For instance, people evaluate more positively stimuli or people they approach as compared to those they avoid (Cacioppo et al., 1993; Kawakami et al., 2007; Wiers et al., 2011; Slepian et al., 2012; Woud et al., 2013b). As a result, approach and avoidance regulate individual-environment interactions through a cyclical loop: continuous evaluation guides behavior appropriately and, in turn, ongoing behavioral activity spurs compatible evaluative processes. This cyclical influence possesses a functional value as it allows individuals to effectively pursue their actions until goal attainment (Förster et al., 2007).

As humans are social organisms endowed with a high level of complexity, they tend to deploy their approach-avoidance repertoire flexibly (Schneirla, 1959). Thus, the interplay between evaluated stimuli and approach-avoidance actions is not hard-and-fast but flexible and context-sensitive. Among other examples, the presence/absence of affective evaluation goals as well as the action outcome moderate the influence of approach-avoidance actions on evaluations (Cacioppo et al., 1993, 2018). Moreover, approach-avoidance orientations may support distal goals, meaning that evaluations can trigger incompatible behaviors (e.g., approaching a very critical researcher) if they ultimately lead to compatible effects (e.g., the exchange will benefit one’s work; Krieglmeyer et al., 2011).

Obviously, approach-avoidance orientations represent the key elements of an adaptive process at the evaluation-behavior interface. Such a process implies a constant combination of sensorimotor interactions with the world involving the brain, the body and the situation. It appears thus compelling to conceptualize approach-avoidance orientations by capitalizing on a view of cognition that emphasizes the role of brain-body-environment interactions.

Historically, since the advent of the cognitivist revolution, cognition has been considered to involve a relatively independent brain system performing computations on abstract and amodal representations (i.e., involving the symbolic translation of perceptual, motor and introspective states). Within this computationalist tradition, approach and avoidance were considered as amodal action representations and the body was a mere vehicle executing those actions based, for instance, on their threshold activation (Bower, 1981; Carver and Scheier, 2000). It has been argued since, that such a view of cognition cannot be adaptive as it is far too rigid and detached from ongoing brain-body-environment interactions, and these objections set the stage for alternative views.

A grounded view of cognition offers a more encompassing account of the flexible two-way influence between approach-avoidance tendencies and evaluation than the computationalist view. From such a perspective, human cognition is *grounded*^1^ in modality specific systems, in the body and actions, as well as in the physical and the social environment (Wilson, 2002; Niedenthal et al., 2005; Pecher and Zwaan, 2005; Barsalou, 2008, 2015). According to one common approach within this perspective, as individuals interact with their world, the brain captures and integrates traces of perceptual, motor and introspective states into multimodal and situated representations (situated conceptualizations, Damasio, 1989; Barsalou, 1999, 2008; Barsalou, 2003, 2015; Versace et al., 2014). A matching between actual experience and some previously captured traces can reactivate the (whole) patterns of traces of the corresponding past experiences. This multimodal simulation aligns the brain and the body with past experiential states (*re-enactment*) depending on what is relevant for the immediate situation (i.e., physical environment, potential for actions, motivational/emotional states, etc.). This process is adaptive because it enables individuals to both anticipate and adapt their interactions to the world based on their past sensorimotor interactions as well as their actual environment. From a grounded perspective, repeated approach-avoidance interactions with the world entail the accumulation of motor, perceptual and introspective states (including positive and negative ones). Thus approach-avoidance orientations can be defined as the re-enactment of these states which impels to move toward or away (Papies and Barsalou, 2015).

Such a grounded perspective dictates specific operationalizations at the empirical level. Indeed, an optimal approach-avoidance manipulation should enable a close matching between the ongoing experience and past approach-avoidance traces. This depends on the potential of the current setting or situation to activate: (1) *prototypical* (i.e., most representative in terms of memory traces), (2) *multimodal*, as well as, (3) *situated* traces of approach-avoidance experiences (Barsalou, 2003, 2005, 2015; Versace et al., 2014; Papies and Barsalou, 2015). Here, we argue that approach-avoidance operationalizations from previous research (even those which are anchored in a grounded perspective) do not entirely reflect their grounded essence, as they have not systematically and jointly integrated the three aforementioned aspects.

### Trace Prototypicality

Past research frequently operationalized approach-avoidance through arm flexion-extension as people generally flex (vs. extend) their arm to approach (vs. avoid) positive (vs. negative) graspable objects. These operationalizations involved among others: pressing the palm below/above the surface of a table, pulling/pushing a joystick, pressing/releasing a button, etc. (Cacioppo et al., 1993; Wentura et al., 2000; Kawakami et al., 2007; Laham et al., 2014). Others relied on oral muscular contractions resembling deglutition of edible substances (approach) or expectoration of noxious ones (avoidance; Topolinski et al., 2014). However, these two motor-based operationalizations cover a relatively restricted number of approach-avoidance experiences: not all external stimuli can be grasped, nor do they concern oral consumption (Rougier et al., 2018). Instead, whole-body operationalizations are more likely to capture most past approach-avoidance experiences. Among these whole-body operationalizations, we find: upper-body posture/inclination (Galton, 1884; Mehrabian, 1968; Word et al., 1974; Riskind, 1984; Price and Harmon-Jones, 2010), walking steps (Worthington, 1974; Dotsch and Wigboldus, 2008; Koch et al., 2009; Fayant et al., 2011; Stins et al., 2011), and simulation of whole-body movements (from a third-person perspective^2^, De Houwer et al., 2001; or from a first-person perspective, Rougier et al., 2018).

### Trace Multimodality

Some scholars constrained operationalizations of approach-avoidance to a single modality (e.g., motor information, Cacioppo et al., 1993; Topolinski et al., 2014; visual information, De Houwer et al., 2001; Rougier et al., 2018). From a grounded perspective, it is indeed conceivable that information in one modality activates other modality-specific traces of approach-avoidance (Damasio, 1989; Barsalou, 1999; Versace et al., 2014). However, an efficient simulation of approach-avoidance states should involve as many different multimodal traces of past experiences as possible (Labeye and Versace, 2007). For instance, a (visual) zoom effect has been combined to the (motor) pulling/pushing joystick movements in order to enhance the operationalization of approach-avoidance orientations (Rinck and Becker, 2007; Krieglmeyer and Deutsch, 2010). Hence, approach-avoidance operationalizations that combine motor, visual and proprioceptive information are more likely to enable the re-enactment of the corresponding states. Among these multimodal operationalizations, we consider: upper-body postures (Price and Harmon-Jones, 2010) or walking steps (Fayant et al., 2011; Stins et al., 2011; Bouman and Stins, 2018). Indeed, these whole-body approach-avoidance behaviors inherently entail changes in information flow and visual perspective while concurrently engaging motor components.

### Trace Situatedness

The majority of work relied on operationalizations of approach-avoidance experiences that scale down the situation to isolated and minimal encounters with stimuli (even when, paradoxically, they make use of prototypical and multimodal aspects, Fayant et al., 2011, Exp. 2; Rougier et al., 2018)^3^. Undoubtedly, this practice runs counter the assumption that the perceptual, motor and introspective traces of approach-avoidance states are not stored in isolation but together with traces of the situation settings in which these states occurred (e.g., elements of the environment, action possibilities, individuals’ intentions, emotional states; Barsalou, 2003, Papies and Barsalou, 2015). Failures to take into account this situatedness may lead to unsatisfactory or ambiguous operationalizations (Markman and Brendl, 2005; Seibt et al., 2008; Van Dantzig et al., 2008; Beatty et al., 2016). Indeed, depending on the situation, the very same muscular contraction can either be considered as approach or avoidance: for example bringing a cake closer or withdrawing one’s hand from a spider both involve arm flexion^4^, and deglutition involves the swallowing of appetitive food stimuli but could also be involved in stress reactions (Ritz and Thöns, 2006). Moreover, as any situation, the experimental setting offers specific action possibilities (i.e., *affordances*) that may interfere with traces targeted by the operationalization of approach-avoidance orientation (Cesario et al., 2010). For instance, intrinsically social stimuli as faces generally evoke whole-body approach-avoidance behavioral actions which are relevant for social interactions. In front of such stimuli, arm flexion-extension operationalizations that activate traces of approach-avoidance experiences in response to graspable objects (Kawakami et al., 2007, 2012, but see Streicher and Estes, 2016) seem unwarranted, to say the least. Therefore, an optimal manipulation of approach-avoidance should rely on contextualized and ecological whole-body approach-avoidance experiences which by virtue of their situatedness re-enact more fully the corresponding states. As appropriate examples of situated approach-avoidance operationalizations we can readily identify those that rely on real life settings and/or confederates (Word et al., 1974; Worthington, 1974)^5^ and those that rely on Virtual Reality (Bailenson et al., 2003; Dotsch and Wigboldus, 2008; Ruggiero et al., 2017) although these works dealt more with proxemics than approach-avoidance behaviors *per se*.

From this literature review, it follows that operationalizations of approach-avoidance orientations relying on **multimodal interactive and contextualized whole-body movements** are the most suitable to reflect their grounded essence. So far, and despite some promising attempts, approach-avoidance operationalizations did not jointly consider the prototypicality, multimodality and situatedness requirements that emerge from an analysis of grounded cognition.

## Overview of the Studies

In this paper, we argue that even if a grounded view of approach-avoidance orientations has gained in popularity over the past few years, somewhat ironically, its theoretical assumptions have not been systematically and jointly considered at the time of choice of operationalization. Bearing in mind that approach and avoidance orientations are grounded in sensorimotor interactions with the physical and social environment, we tentatively propose a prototypical, multimodal and situated operationalization. An appropriate and exhaustive operationalization of approach-avoidance orientations is crucial as this constitutes one of the major obstacles when connecting theory to data (Rakover, 1981). To assess the viability of this operationalization, we implemented it in the examination of the influence of approach-avoidance behaviors on interpersonal evaluations. In all studies, we manipulated approach-avoidance orientations through ecological whole-body approach-avoidance behaviors and measured evaluations in a self-reported way. As a general hypothesis, and in line with previous literature (Cacioppo et al., 1993; Slepian et al., 2012), we anticipated that, using highly ecological settings, approach behaviors would lead to more positive evaluations as compared to avoidance behaviors. We followed a two-stage process to test this hypothesis and incrementally consider the grounded assumptions. In a first stage, in order to provide continuity with past research, we relied on operationalizations that have been previously used in the literature (but not in the field of interpersonal evaluations) and that satisfied the prototypicality and the multimodality requirements: upper-body incline/posture. We set these behaviors in the context of social interactions as we deem them particularly relevant for this kind of situation and tested their effect on interpersonal evaluations in four pilot studies. In the second stage, and in a break with past research, we went further in the situatedness consideration and took seriously the grounded nature of approach-avoidance orientations. To this aim, we relied on upper-body incline and walking steps operationalizations in two main studies that we conducted through immersive virtual reality (VR). VR is increasingly viewed as a promising tool in the study of social interactions in that it allows considering the ongoing individual-environment interaction while maximizing experimental control (Blascovich et al., 2002; McCall, 2015; Pan and Hamilton, 2018). In all studies we planned to run at least 50 participants per condition as recommended by Simmons et al. (2013). Such a criterion enabled us to detect an effect size *η*^2^ comprised between 0.05 and 0.15 (depending on the design) with a power of 80%. We collected and analyzed anonymously all data with written informed consent from participants in accordance with the American Psychological Association’s ethical principles. However, we did not seek the explicit ethics approval as it was not required for the present studies as per Université de Paris’s guidelines and applicable national regulations.

## Pilot Studies

As an initial step in considering the grounded nature of approach-avoidance orientations in their operationalization we conducted four pilot studies. In these pilots, we aimed at replicating and extending the influence of approach-avoidance orientations on self-reported evaluations relying on prototypical and multimodal operationalizations by adapting existing inductions: upper-body incline/posture. We set these behaviors in the context of a social interaction (i.e., face stimuli). By doing so, we intended to maximize trace activation and expected more positive evaluations in the approach than in the avoidance condition. The procedure was comparable throughout the pilots: participants evaluated faces while performing an approach or avoidance behavior. At the end, they also indicated to what extent they found the task pleasant, difficult and tiring to control for any potential confounded variables. We present the main elements of the pilot studies below and provide details for these pilots in Supplementary Material 1.

In Pilot 1 (*N*_Analyzed_ = 50), participants were seated between two wooden boards perpendicular to which we affixed two computer mice and facing a computer screen (see Supplementary Material 1). Pretexting a study on ergonomic positions, we asked them to greet computerized faces (taken from Oosterhof and Todorov, 2008) while performing different movements. Depending on the block of a within-participants design, participants had to either lean their upper-body forward or backward in order to click the corresponding mouse button (behind vs. in front of the coronal plane). The mouse click triggered the appearance of a speech bubble saying “hello,” indicating that participants effectively greeted the character. After this instrumental movement (i.e., greeting), participants returned to the body’s “home” position (i.e., an upright position) and rated the pleasantness of the face (from 1: *very unpleasant* to 7: *very pleasant*).

In Pilot 2 (*N*_Analyzed_ = 107), we relied on a between-participants design. We further added contextual cues by connecting an upper body to each face and placing them in an office room background. These were projected real size on a wall. We also reduced the distance between the wooden boards to obtain a more ecological movement amplitude. Pretexting a study on impression formation during a job-interview, we asked participants to greet characters verbally while leaning their upper-body either forward or backward depending on condition. In order to circumvent the fact that both approach and avoidance movements were performed before evaluating characters (as this could have been potentially an issue in the case of the manipulation in Pilot 1) participants had to maintain the position while evaluating characters. Instead of asking participants to judge the faces, we asked them to provide their impression of them on a scale anchored at −3: *I do not like at all* and +3: *I like very much* (Chen et al., 2004).

In Pilot 3 (*N*_Analyzed_ = 97), we manipulated approach-avoidance orientations through corresponding postures and relied on the same stimuli as in Pilot 2. Participants were seated in front of a computer screen and were instructed to give their impression of characters verbally, while leaning forward or backward throughout the experimental procedure.

Pilot 4 (*N*_Analyzed_ = 154) followed the same procedure as Pilot 3 except two changes. To increase reliance on their affective feeling, we led participants to believe that they subliminally received pseudo-individualizing information about each presented target-person (Yzerbyt et al., 1998). To increase ecological validity, we also sampled pictures instead of computerized faces from a distinct database (i.e., the Chicago Face Database, see Ma et al., 2015).

Across the four pilots, we failed to show a positive effect of approach behaviors (as compared to avoidance) on interpersonal evaluations. A random effects mini meta-analysis (with the “metafor” R package) on the standardized regression coefficients (Kim, 2011) revealed a statistically non-significant effect of approach-avoidance behaviors on evaluations, *z* = −0.75, *p* = 0.455, *β*_Z_ = −0.05, 95% CI (−0.17, 0.07)^6^.

Upper-body inclination/postures used in previous research are arguably prototypical and multimodal operationalizations of approach-avoidance orientations, which are also relevant in the context of face evaluation. However, such operationalizations only partially consider the grounded essence of approach-avoidance orientations as they are low in situatedness. The social interaction context and face stimuli may have not been sufficiently interactive to satisfy the situatedness requirement and allow for the re-enactment of approach-avoidance experiences. With an objective of bringing a possible solution with respect to this aspect, we used VR − an immersive and interactive tool − in the two following studies.

## Main Studies: A Virtual Reality Setting

In Study 1 and 2, we tested the effect of approach-avoidance behaviors on interpersonal evaluations relying on VR and using self-reported evaluations. We expected more positive evaluations in the approach than in the avoidance condition, with the control condition falling in between. Importantly, the inconclusive results of the four pilot studies may also be due to the failure of activating approach-avoidance tendencies. Thus, to directly address this issue in these studies we also included additional measures of approach-avoidance tendencies in order to assess the construct validity of the manipulation. We thus measured action tendencies (with the Visual Approach/Avoidance by the Self Task, VAAST; Rougier et al., 2018, for a similar procedure see Smith and Bargh, 2008) and the activation of approach-avoidance neuropsychological systems (Reinforcement Sensitivity Theory of Personality Questionnaire, RST-PQ; Corr and Cooper, 2016). We expected that our manipulation of approach-avoidance orientations would activate the corresponding action tendency and neuropsychological system. We also took care to measure the feeling of Presence and Cybersickness that could hinder the Virtual Reality experience (see Pan and Hamilton, 2018), as well as the judgment of pleasantness, tiredness and difficulty of the task to control for any potential confounded variables. All hypotheses, measures, instructions and statistical analyses were pre-registered^7^,^8^.

### Study 1

#### Methods

##### Participants

In total, 211 French-speaking participants took part in the study in exchange of partial credit course or 15€. They were randomly assigned to the approach, avoidance, or control conditions in a between-participants design^9^. We excluded participants that: guessed the hypothesis (5), did not follow the instructions (e.g., using only the head instead of the upper body; 56) and reported having consumed substances (3). Finally, we excluded one participant with excessive missing data (46.67%) due to a technical problem with the VR equipment. We thus analyzed the data of the remaining 162 participants.

##### Material

Twelve first names per gender, half of them containing the sound /o/ (e.g., Margaux, Jerome) and the other half containing the sound /i/ (e.g., Emeline, Remy) served as stimuli for the VAAST. We controlled them for frequency based on the national database (Institut National de la Statistique et des Etudes Economiques [INSEE], 2015).

##### Procedure

###### Virtual reality task

Upon their arrival, participants received instructions about the VR task on a computer screen. The task was presented as a study on impression formation and administered through a VR headset (HTC Vive^©^). Participants were seated at a table in a neutral virtual room and had to maintain an upright position. Each virtual character sat in front of them and greeted them by saying “hello.” Depending on the condition, participants had to reply back “hello” and perform a 10-degree forward-lean (approach condition), a 10-degree backward-lean (avoidance condition) or no movement (control condition). A Likert-type scale appeared in the virtual environment 2000 ms after participants performed the correct action. While maintaining their position, participants used the HTC controllers to provide their impression of the character anchored at 1 (negative) and 7 (positive). Once the response was recorded, the virtual character walked away and participants in the approach and avoidance conditions were instructed to go back to the central position. Then, participants waited for the appearance of the next virtual character to repeat the sequence. After five training trials with a test character, participants encountered 30 characters (15 men and 15 women). In line with previous research, we expected more positive evaluations in the approach than in the avoidance condition.

**FIGURE 1:**
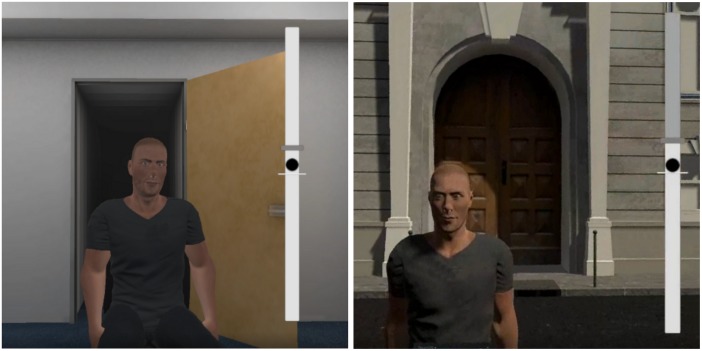
Image captures from the Virtual Reality task in Study 1 (left) and Study 2 (right).

Based on our theoretical rationale, we refrained from explicitly mentioning approach or avoidance labels in the instructions in order to limit potential demand characteristics and the direct influence of these labels on evaluations (Van Dessel et al., 2015). Thus, in order to assist participants in reaching the correct orientation without an explicit mention of the terms “approach” or “avoidance,” we presented them a position bar displaying the onset position (the white mark on Figure 1), the requested final position (the gray mark on Figure 1) and their tracked position (the black circle on Figure 1) on the right side of the screen. Using this position bar, their task was to align their upper-body to the requested position. If participants deviated too much from the requested position, they received an auditory feedback.

##### Action tendencies

After the VR task, participants performed the VAAST (Rougier et al., 2018) to check if our manipulation of approach-avoidance orientations activated the corresponding action tendency. They had to categorize first names depending on the sound they contained (i.e., the /o/ vs. /i/ sound) by pressing a “move forward” key (approach response) or a “move backward” key (avoidance response). In one block, participants had to approach first names containing the sound /o/ and avoid those containing the sound /i/. In the other block, this was reversed. Each trial began with a white circle displayed in the center of the screen prompting participants to press a “start” button. Then, a fixation cross was displayed (with a random duration of 800–2000 ms) and participants had to keep their finger pressed until a first name appeared. When the target name appeared, participants had to categorize it by pressing the “move forward” or “move backward” key four times, as quickly and as accurately as possible. Depending on keypress, the background image and the target first name was zoomed in (i.e., “move forward” button, approach) or zoomed out (i.e., “move backward” button, avoidance) by 10% after each button press. In each block, participants performed 8 training trials followed by 48 experimental trials. We recorded reaction time (RT) at the onset of the name until the first keypress. At the outcome, participants indicated their age, gender, laterality and if they were fluent in French (in case they were not, they indicated their skills on a scale from 1 = *very low level* to 7 = *very high level*). We expected participants to approach stimuli faster in the approach than in the avoidance condition but to avoid stimuli faster in the avoidance than in the approach condition, or to put it short an interaction between movement and response type.

##### Neuropsychological systems

Then, participants completed the French version of the RST-PQ (Corr and Cooper, 2016; L.-C. Vannier, personal communication, December 4, 2017) to check if our manipulation of approach-avoidance orientations activated the corresponding system. Based on the revised reinforcement sensitivity theory (Corr and McNaughton, 2012), this questionnaire measures the Behavioral Approach System (BAS, related to approach behaviors and appetitive stimuli; 29 items), the Fight-Flight-Freeze System (FFFS, related to active avoidance behaviors and aversive stimuli; 10 items) and the Behavioral Inhibition System (BIS, related to passive avoidance behaviors and conflictual stimuli; 15 items). The RST-PQ has the advantage of taking into account the multidimensionality of the BAS and distinguishing the FFFS from the BIS. We expected higher BAS scores in the approach than in the avoidance condition and higher FFFS scores in the avoidance than in the approach condition.

##### Complementary measures

Subsequently, participants completed the French versions of the Presence Questionnaire (PQ; Witmer and Singer, 1998; Robillard et al., 2002) and the Simulator Sickness Questionnaire (SSQ; Kennedy et al., 1993; Bouchard et al., 2007). They also indicated to what extent they found the VR task pleasant, difficult and tiring (on a scale from 1 = *not at all* to 7 = *extremely*). All these complementary measures were included to control for any potential confound. Finally, they reported any trouble or substance intake which could have impaired their performance. They were probed for suspicion, debriefed and compensated for their participation.

#### Results

We ran several General Linear Model analyses. In order to test the linear effect of movement, we created two contrast codes. In the first, we opposed the approach (+1) to the avoidance condition (−1), ignoring the control condition (0). In the second, we opposed the control condition (+2) to both the approach (−1) and avoidance conditions (−1). As participants judged the task more tiring in the avoidance than in the approach condition (*M*_Approach_ = 2.60, *SE*_Approach_ = 0.23; *M*_Avoidance_ = 3.45, *SE*_Avoidance_ = 0.21; *F*(1, 155) = 7.47, *p* = 0.007, *β*_Z_ = −0.26, 95% IC [−0.45, −0.07]), we included the tiredness judgment in the analysis to control for this potential confound^10^. All reported descriptive statistics were those estimated by the models and the 95% confidence intervals reported hereafter are based on the standardized differences between the tested means.

##### Evaluations

We deleted trials where participants performed a wrong movement (1.30%), deviated from the position they had to maintain (6.32%) and/or did not directly reach the correct position (5.13%). On the remaining trials, we estimated a linear mixed-effects model with the linear codes of contrast, tiredness judgment and their interactions terms as fixed factors as well as participants and stimuli as random factors (with the “lmer” R package). Contrary to the tested hypothesis, the first contrast revealed that evaluations did not significantly differ between the approach (*M* = 4.26, *SE* = 0.17) and the avoidance conditions (*M* = 4.34, *SE* = 0.16), *F*(1, 153.09) = 0.39, *p* = 0.534, *β*_Z_ = −0.03, 95% IC [−0.12, 0.06]. No other effect was significant, *F*s < 1.39, all *p*s > 0.239.

##### Action tendencies

Concerning the VAAST, we examined RTs for experimental trials only and removed incorrect trials (3.29 %). In order to correct a positively skewed distribution, we deleted RTs faster than 200 ms or above 2000 ms (1.06%) and applied a log-transformation on raw RTs. We estimated a linear mixed-effects model with the linear codes of contrast, response type (approach, avoidance), tiredness judgment and their interaction terms as fixed factors, as well as participants and stimuli as random factors (with the “lmer” R package). The analysis first revealed a significant main effect of response type, *F*(1, 14360) = 62.81, *p* < 0.001, *β*_Z_ = −0.11, 95% IC [−0.14, −0.08]. Participants were faster to approach (*M* = 719.10 ms^11^, 95% IC [699.24, 739.52]) than to avoid (*M* = 741.74 ms, 95% IC [721.26, 762.80]) the first names. However, we did not obtain the expected interaction between the first contrast and response type, *F*(1, 14360) = 0.06, *p* = 0.806, *β*_Z_ = 0.00, 95% IC [−0.03, 0.04]: participants were not faster to approach (vs. avoid) first names in the approach than in the avoidance condition (see Table 1). The analysis also revealed a marginal interaction between response type and tiredness judgment indicating that the more participants judged the task as tiring, the quicker they were to approach than to avoid, *F*(1, 14360) = 3.24, *p* = 0.02, *β*_Z_ = −0.02, 95% IC [−0.03, 0.00].

**TABLE 1:** Estimated means and standard errors (or confidence intervals) for evaluations, neuropsychological systems and action tendencies.

	**Avoidance**		**Control**		**Approach**	
**Variable**	***M***	***SE (or 95 % CI)***	***M***	***SE (or 95 % CI)***	***M***	***SE (or 95 % CI)***
**Evaluations**						
Pilot 1^a^	3.54	0.15	*/*	/	3.78	0.16
*Bloc 1*	3.7	0.21	*/*	/	3.44	0.36
*All data*	3.54	0.15	*/*	/	3.78	0.16
Pilot 2^b^	0.35	0.12	*/*	/	0.11	0.12
Pilot 3^b^	0.13	0.2	*/*	/	0.23	0.19
Pilot 4^b^	0.45	0.13	0.4	0.13	0.56	0.14
Experiment 1^a^	4.34	0.16	4.31	0.16	4.26	0.17
Experiment 2^a^	4.46	0.17	4.41	0.17	4.34	0.17
**Neuropsychological Systems**						
BAS						
Experiment 1	2.89	0.05	2.92	0.05	2.84	0.05
Experiment 2	2.93	0.05	2.97	0.05	2.96	0.04
FFFS						
Experiment 1	2.12	0.08	2.01	0.07	2.07	0.08
Experiment 2	2.17	0.08	2.18	0.08	2.16	0.07
**Action Tendencies**						
Approach RT						
Experiment 1	720.54	[689.52, 753.70]	732.89	[702.11, 765.10]	704.16	[672.50, 736.57]
Experiment 2	731.43	[701.35, 762.04]	750.7	[719.82, 782.11]	739.52	[710.52, 769.70]
Avoidance RT						
Experiment 1	744.71	[711.94, 778.99]	755.21	[723.43, 787.61]	725.6	[692.98, 759,76]
Experiment 2	752.95	[722.70, 785.25]	782.9	[750.70, 815.66]	762.04	[732.16, 793.14]

*M, estimated mean; SE, estimated standard error; CI, confidence interval; BAS, behavioral approach system; FFFS, fight, flight and freeze system; RT, reaction times. ^*a*^Scale from 1 to 7. ^*b*^Scale from −3 to +3.*

##### Neuropsychological systems

For RST-PQ scores, we estimated a linear regression model with the two contrast codes, tiredness judgment and their interaction terms as predictors. Contrary to what we expected, we did not obtain higher BAS scores in the approach than in the avoidance condition, *F*(1, 151) = 0.46, *p* = 0.499, *β*_Z_ = −0.06, 95% IC [−0.25, 0.12]. The results are even in the opposite direction with higher BAS scores in the avoidance (*M*_BAS_ = 2.89, *SE*_BAS_ = 0.05) than in the approach condition (*M*_BAS_ = 2.84, *SE*_BAS_ = 0.05). Neither we obtained higher FFFS scores in the avoidance (*M*_FFFS_ = 2.12, *SE*_FFFS_ = 0.08) than in the approach condition (*M*_FFFS_ = 2.07, *SE*_FFFS_ = 0.08), *F*(1, 151) = 0.21, *p* = 0.649, *β*_Z_ = −0.04, 95% IC [−0.23, 0.14] although the pattern was in the expected direction. There was no other significant effect, nor for the BAS, neither for the FFFS, *F*s < 2.68, *p*s > 0.104.

#### Discussion

In Study 1, we took advantage of the immersive and interactive nature of VR to implement a grounded operationalization of approach-avoidance orientations and to test their effect on interpersonal evaluations. However, we failed to show the expected positive influence of approach on evaluations. We also did not obtain any indication of an activation of the corresponding action tendencies or neuropsychological systems. Nevertheless, as a relatively substantial part of the sample did not correctly perform the instructed action, it appears that upper-body incline was not very intuitive to participants within this setting. This may have rendered the operationalization of approach-avoidance orientations ambiguous. In Study 2, we pursued the examination and relied on an experimental variation of the foregoing grounded operationalization.

### Study 2

#### Methods

##### Participants

Two-hundred and four participants took part in the study in exchange of partial credit course or 15€. They were randomly assigned to the approach, avoidance, or control conditions in a between-participants design. We excluded participants that: guessed the tested hypothesis (2), did not follow the instructions (e.g., steps incompletely done; 7), reported substance intake (4) and declared low French skills (i.e., below 5 on the 1 to 7 scale; 1). Due to an experimenter error, one participant received opposite behavioral instructions from the behavior he had to perform in VR. We excluded this participant and analyzed the data of the remaining 189 participants.

##### Procedure

We followed exactly the same procedure as in Study 1, except the approach-avoidance orientations operationalization. This time, participants stood at a bus stop in a virtual street and had to maintain an upright position (Figure 1). Virtual characters came across to them and greeted them by saying “hello.” Depending on the condition, participants had to reply back “hello” making one step (approx. 20 cm wide) forward (approach condition), backward (avoidance condition) or standing in place (control condition).

#### Results

Again, we ran several General Linear Models to test our predictions. We created the same two contrast codes as in Study 1 in order to test the linear effect of movement. In the first, we opposed the approach (+1) to the avoidance condition (−1), ignoring the control condition (0). In the second, we opposed the control condition (+2) to both the approach (−1) and avoidance conditions (−1).

##### Evaluations

We deleted trials where participants performed a wrong movement (0.20%), deviated from the position they had to maintain (1.85%) and/or did not directly reach the correct position (2.4%). On the remaining trials, we estimated a linear mixed-effects model with the same linear codes of contrast as fixed factors as well as participants and stimuli as random factors (with the “lmer” R package). Again, the analysis revealed that evaluations did not significantly differ between the approach (*M* = 4.34, *SE* = 0.17) and the avoidance condition (*M* = 4.46, *SE* = 0.17), *F*(1, 186.43) = 1.03, *p* = 0.310, *β*_Z_ = −0.04, 95% IC [−0.13, 0.04]. The second contrast also was not significant, *F* < 1, *p* = 0.922.

##### Action tendencies

Concerning the VAAST, we examined RTs for experimental trials only and removed incorrect trials (3.72 %). In order to correct a positively skewed distribution, we deleted RTs faster than 200 ms or above 2000 ms (1.06%) and applied a log-transformation to raw RTs. We estimated a linear mixed-effects model with the linear contrast, response type (approach, avoidance) and their interaction terms as fixed factors as well as participants and stimuli as random factors (with the “lmer” R package). As in Study 1, participants were faster to approach (*M* = 740.26 ms, 95% IC [721.26, 759.76]) than to avoid (*M* = 765.86 ms, 95% IC [746.20, 786.03]) the first names, *F*(1, 17050) = 93.24, *p* < 0.001, *β*_Z_ = −0.12, 95% IC [−0.15, −0.10]. We did not obtain the expected interaction between the first contrast and response type, *F*(1, 17050) < 0.01, *p* = 0.962, *β*_Z_ = −0.00, 95% IC [−0.03, 0.03] (see Table 1).

##### Neuropsychological systems

For RST-PQ scores, we estimated a linear regression model with the two contrast codes as predictors. The analysis revealed no effect of the approach-avoidance orientations manipulation on BAS scores (*M*_Approach_ = 2.96, *SE*_Approach_ = 0.04; *M*_Avoidance_ = 2.93, *SE*_Avoidance_ = 0.05, *F*(1,182) = 0.14, *p* = 0.705, *β*_Z_ = 0.03, 95% IC [−0.14, 0.21]) neither on FFFS scores (*M*_Approach_ = 2.16, *SE*_Approach_ = 0.07; *M*_Avoidance_ = 2.17, *SE*_Avoidance_ = 0.08, *F*(1, 182) < 0.01, *p* = 0.962, *β*_Z_ = −0.00, 95% IC [−0.18, 0.17]).

#### Discussion

In Study 2, although we increased the ecological character and situatedness of the operationalization of approach-avoidance orientations, we again failed to confirm the theorized prediction. Approach-avoidance behaviors did not influence evaluations as well as the activation of corresponding action tendencies or neuropsychological systems.

### Complementary Analyses

Although VR is a promising tool to operationalize approach-avoidance as grounded in individual-world experiences, it nevertheless remains a technology-mediated experience. Thus, virtual approach-avoidance interactions might enable to re-enact internal states only when individuals did not consciously perceive such a mediation (Parsons and Rizzo, 2008). That is, in the case the virtual environment successfully supports approach-avoidance interactions while offering the same sensorimotor information as in non-virtual settings and providing individuals the feeling of “being there.” This subjective experience of being in one environment, even when one is physically situated in another, is coined the “feeling of *presence*” (Witmer and Singer, 1998). Some scholars consider the feeling of presence as reflecting the full integration of every relevant aspect of the situation pertaining to the “here and now” including: movement and perception, actions, representation of the self in the overall situation, possibilities for action, etc. (Carassa et al., 2005; Riva, 2009; Mennecke et al., 2011; Riva and Waterworth, 2014; Willans et al., 2015). In this sense, the notion of presence may gauge the extent to which cognition is grounded in the virtual environment, and may be a necessary condition to re-enact approach-avoidance states through VR.

The overall feeling of presence in the current studies (*M*_Exp1_ = 95.52, *SE*_Exp1_ = 1.07; *M*_Exp2_ = 92.75, *SE*_Exp2_ = 1.05) was lower than the French speaking norm (*M* = 104.39, *SE* = 1.89; from the Cyberpsychology Lab at University of Quebec in Outaouais, 2013). This moderately low feeling of presence could explain that we failed to obtain the positive effect of approach on evaluations. For this reason, we added the feeling of presence as a fixed factor in the models previously estimated. For the sake of clarity, we only report results that we deemed relevant for the goal of this paper (the interested reader can refer to Supplementary Material 2).

#### Complementary Analyses of Study1

Although not significant, the patterns showed that the more participants felt being present in the situation the more the approach manipulation activated the BAS as compared to the avoidance condition, *F*(1, 144) = 0.36, *p* = 0.549, *β*_Z_ = 0.00, 95% IC [−0.01, 0.02]^12^. However, the patterns also showed that the more participants felt being present the less the avoidance manipulation activated the FFFS as compared to the approach condition, *F*(1, 144) = 1.11, *p* = 0.295, *β*_Z_ = 0.01, 95% IC [−0.01, 0.02].

A closer inspection of evaluative ratings suggested that the more participants felt being present in the situation, the more they evaluated positively the characters in the avoidance as compared to the approach condition, although this effect was not significant, *F*(1, 146.3) = 0.04, *p* = 0.847, *β*_Z_ = −0.00, 95% IC [0.00, 0.01].

#### Complementary Analyses of Study 2

The patterns reveal that the more participants felt being present in the situation the more the approach manipulation activated the BAS compared to the avoidance condition, *F*(1, 179) = 3.95, *p* = 0.048, *β*_Z_ = 0.01, 95% IC [0.00, 0.02]. Correspondingly, the more participants felt being present in the situation the more the avoidance manipulation activated the FFFS compared to the approach condition, *F*(1, 179) = 1.49, *p* = 0.224, *β*_Z_ = −0.01, 95% IC [−0.02, 0.00], although the latter results were not significant.

Interestingly, including presence in the analysis of evaluative ratings revealed that the more participants felt being present in the situation, the less they evaluated positively the characters in the avoidance as compared to the approach condition, *F*(1, 180.9) = 0.20, *p* = 0.66, *β*_Z_ = −0.00, 95% IC [−0.00, 0.01].

#### Discussion of Complementary Analyses

These exploratory analyses suggest that the approach-avoidance manipulation is contingent on the way participants experience the immersive virtual situation. At least in Study 2, the analyses revealed patterns of interaction between the manipulation of approach-avoidance orientations and the feeling of presence on the activation of the neuropsychological systems. Indeed, the corresponding motivational states seem to be activated by the manipulation when individuals felt being present (in a non-mediated interaction with the environment). Although non-anticipated, we deem these results important as they emphasize the role of ongoing individual-environment interaction in social cognition and arguably fit well with a grounded view of cognition putting subjective sensorimotor experiences at the core of knowledge. However, the results of Study 1 are less clear with patterns of interaction in the opposite direction. As previously mentioned, a large proportion of participants had not correctly performed the requested action in Study 1, while this was not the case in Study 2. This may suggest that upper-body incline was a more ambiguous operationalization of approach-avoidance experiences than walking and may explain the mitigated pattern.

## General Discussion

In this paper, our aim was to capitalize on a grounded view of cognition to develop a thorough and appropriate operational definition of approach and avoidance. According to this view, an optimal operationalization should enable a close matching between ongoing experience and past approach-avoidance traces. To this aim, we relied on prototypical whole-body movements, involving multi-sensory information, in relevant interpersonal contexts. We implemented these operationalizations in the study of the influence of approach and avoidance on interpersonal evaluations. In six studies, we relied on prototypical and multimodal operationalizations previously used in approach-avoidance studies (e.g., evaluative-assimilation, Fayant et al., 2011; cognitive categorization, Price and Harmon-Jones, 2010). In the last two studies, we went a step further and relied on immersive VR in order to fully consider the grounded aspect of approach-avoidance orientations. Doing so, we also satisfied a third (and frequently overlooked) requirement for an optimal grounded operationalization of approach-avoidance: its situatedness. Despite this, the present studies failed to show more positive evaluations in the approach than in the avoidance condition. Including all standardized regression coefficients from VR studies and pilots in a random effects meta-analysis revealed a statistically non-significant effect of approach-avoidance behaviors on evaluations, *z* = −1.06, *p* = 0.2887, *β*_Z_ = −0.03, 95% CI (−0.07, 0.02)^13^. This estimated effect is even in the opposite direction with more negative evaluations in the approach than in the avoidance condition. Thus, in the present studies, it seems as if approach and avoidance do not influence interpersonal evaluations. This non-finding is puzzling and opposes a wealth of studies that obtained reliable effects of approach-avoidance actions on evaluations (Cacioppo et al., 1993; Slepian et al., 2012; Woud et al., 2013b).

With all cautions taken, the fact that the influence of approach-avoidance on evaluations did not emerge with the use of more ecological behavioral operationalizations raises some questions. First, it may be the case that previous effects were only the fact of unimodal and decontextualized operationalizations of approach-avoidance experiences that activated a very specific and limited pattern of traces. However, social psychologists have the ultimate goal of studying how human social cognition unfolds in daily individual-environment interactions, rather than in (overly) simplistic approximations of those situations (e.g., being seated in front of pictures or words presented on a computer screen in an experimental box). In isolated and simplistic situations, a very narrow and specific pattern of traces may be activated. However, when common sensory surroundings stimulate the individuals’ body and brain, the same pattern may interact with others and become highly context-dependent. In line with this, Varela et al. (1991, p. 94) observed that “the brain is a highly cooperative system: the dense interconnections among its components entail that eventually everything going on will be a function of what all the components are doing.” Moreover, the effects of approach-avoidance tendencies on evaluations are often studied for intervention purposes (e.g., addiction treatment, Wiers et al., 2011; prejudice reduction, Kawakami et al., 2007; phobia reduction, Jones et al., 2013). Nevertheless, the effectiveness of interventions would be very limited if daily life experiences differ from the traces involved in these specific intervention phases.

Second, the present studies differed in some aspects from previous work. For instance, we asked participants to evaluate individuals after each encounter while many research involved evaluations only after the presentation of the stimulus set. While the former may be considered as a “priming paradigm,” the latter resembles more a learning paradigm (Gast et al., 2012; Laham et al., 2014). Moreover, in previous literature participants are often required to repeatedly approach and avoid specific stimuli/categories, unlike the procedure we relied on in this paper. Thus, extensive behavioral repetition may be necessary to obtain effects of ecological approach-avoidance behaviors on evaluation. It may also be necessary to perform both approach and avoidance behaviors contingent upon specific stimuli/categories. Indeed, according to a grounded perspective, these contingencies could foster the integration of multimodal traces of ongoing experiences (Barsalou, 1999) and/or predictive inferences based on these multimodal representations (Van Dessel et al., 2018b). These observations call for further work along these lines while pursuing the use of ecological operationalizations of approach-avoidance orientations.

Third, in our studies we relied on neutral faces as the effect of approach-avoidance behaviors on evaluations was often studied with neutral stimuli (e.g., neutral ideograms, non-words, fictitious social groups, neutral faces; Cacioppo et al., 1993; Slepian et al., 2012; Van Dessel et al., 2018a). However, the use of such stimuli may have been problematic for two reasons. First, it is possible that neutral expressive faces are not very prototypical of interpersonal approach-avoidance experiences and may thus require more expressive ones. Second, some scholars suggested that approach-avoidance behaviors influence evaluations depending on their motivational compatibility with stimuli: yielding more positive evaluations in the case of compatibility (i.e., approached-positive and avoided-negative), but more negative evaluations in the case of incompatibility (i.e., approached-negative and avoided-positive, Centerbar and Clore, 2006; Krishna and Eder, 2019). This possibility may explain the absence of effects and deserves further investigation. For example, we could add an emotional expression on individuals faces (Dru and Cretenet, 2008; but see Woud et al., 2013a). Current research developments in our lab are specifically dedicated to this issue.

Fourth, as we globally failed to activate approach-avoidance action tendencies and neuropsychological systems, we may have faced a construct validity issue. One or more elements in the situation may have impeded the reactivation of past approach-avoidance traces. For instance, if cognition is grounded in multimodal processes relevant for the immediate situation, the pattern of captured traces would differ depending on the task at stake (Barsalou, 1999). We asked participants to perform ecological interpersonal actions without explicitly labeling them as approach-avoidance. This was done in order to avoid potential demand characteristics and the direct influence of these labels on evaluations. In turn, participants may have been overly focused on understanding and correctly performing the requested action rather than on merely interacting with the characters (as reflected by the large proportion of participants in Study 1 that did not perform the action correctly). This may have led to a different pattern of traces than the one associated with usual interpersonal approach-avoidance experiences.

Finally, in two studies we relied on immersive VR to satisfy the requirements of a grounded perspective in the operationalization of approach-avoidance orientations. However, the use of VR is not without challenges and any asynchrony between the visual (virtual) environment and proprioceptive or motor information may impede individuals’ experience of having a body in the environment as well as their experience of interacting with elements of it (Pan and Hamilton, 2018). If it is indeed the case, traces of previous approach-avoidance experiences may have not been appropriately activated by the ongoing VR experience. Importantly, the exploratory results of the studies suggested the importance of taking into account the quality of the VR experience in the ecological operationalization of approach-avoidance. Indeed, the more individuals felt present in the virtual environment and the more the ecological approach-avoidance behaviors activated the corresponding neuropsychological systems (at least in Study 2). Thus, following others (Pan and Hamilton, 2018), we agree that increasing the feeling of presence is thus the necessary next step (and challenge) in the avenue of research on the ecological operationalization of approach-avoidance orientations through VR.

Beyond these VR issues, the obtained exploratory results may be of theoretical interest. The feeling of presence is not confined to VR but consists in a more general psychological state − similar to a basic state of consciousness (Loomis, 1992) − accompanying all interactions with the physical and social environment, be it real or virtual (i.e., inner presence, Riva et al., 2004; Carassa et al., 2005; Riva, 2009; Willans et al., 2015). Some consider presence as emerging from the match between simulated sensory predictions (i.e., relevant past experiences traces) and the ongoing sensory consequence of an action (i.e., traces captured from the ongoing interaction, Riva et al., 2011). Others regard presence as a dynamical self-organizing system that emerges from a constant interaction between an organism and its environment and can further combine with emotional dynamical systems (Willans et al., 2015). Due to these potential links between presence, action, emotion, intentionality and embodiment, we deem important to further investigate the role of presence in the operationalization of approach-avoidance orientations and their downstream consequences. For instance, future work could test if the feeling of presence is an experiential phenomenon that is either necessary and/or sufficient to manipulate approach-avoidance.

## Conclusion

We believe that the present findings and non-findings are interesting for the topic of this Special Issue as they suggest that approach and avoidance are much more complex phenomena than basic whole-body movements toward or away from a person (or object). Just as other actions, approach and avoidance are rooted in the subjective experience of the ongoing individual-environment interaction (James, 1904). Hence, we view the present work as a first step and a basis for further discussion and research on proper operationalizations of approach-avoidance experiences considered within the realm of a grounded view of cognition. We also believe that this work stimulates new fundamental questions about the influence of approach-avoidance behaviors on evaluations.

## Data Availability

The datasets analyzed and the corresponding R scripts for the pilots can be found in Open Science Framework (https://osf.io/quk3j/?view_only=acca3acd55284968a935b97debf55828). The datasets analyzed and the corresponding R scripts for the two studies can be found in Open Science Framework (https://osf.io/sqhvw/?view_only=9624d0c0d73345029d48a67ea4892e9f).

## Ethics Statement

We collected and analyzed anonymously all data in accordance with the American Psychological Association’s ethical principles. However, we did not seek the explicit approbation of an ethics committee for the present studies as there is no law concerning non-interventional research in France.

## Author Contributions

All authors contributed to the study concept and design. Data collection was organized by IN. She performed the data analysis and was supported by TA and M-PF in the interpretation of the results. All authors drafted the manuscript and approved the final version of the manuscript.

## Conflict of Interest Statement

The authors declare that the research was conducted in the absence of any commercial or financial relationships that could be construed as a potential conflict of interest.

## Footnotes

1 In keeping with Barsalou (2008), we used the term *grounded* rather than *embodied* to address all forms of cognitive grounding: not only based on the body, but also on modalities, on situated actions and on physical as well as social environments.

2 Admittedly, the Manikin Task of De Houwer et al. (2001) does not involve whole-body movements *per se*. In this task, participants have to move a little figure representing the self toward or away from the stimuli. Even if the Manikin Task is not anchored in a grounded perspective, it is still conceivable that the perceived visual distance change could re-enact whole-body approach-avoidance experiences.

3 These experiments involved for example approach-avoidance toward isolated words presented on a screen. Indeed, in everyday life words on posters or signs may sometimes appeal or repel people, but evidently this constitutes a fairly small subset of approach-avoidance experiences.

4 The same contraction can also be interpreted differently across studies. Generally, scholars considered that flexion is involved in bringing something closer to the self (approach) while extension is involved in pushing something away (avoidance). However, some studies operationalized approach as extension and avoidance as flexion (Mertens et al., 2018).

5 Obviously, the use of real life settings or confederates implies a lot of methodological shortcomings (e.g., confederates enter an experimental social interaction with their own past experiences background and perfectly controlling one’s behavior in such situation is nearly impossible to achieve; McCall, 2015).

6 For Pilot 1, we only considered the first block of trials in the meta-analysis. Even if this choice affected power, it was done for the sake of comparability with the other pilots that used a between-participants design and also because there was an interaction between movement and block order.

7 osf.io/sqhvw

8 After extensive consultation among all authors, we applied exclusion criteria deviating from the pre-registered ones. **(1)** Because of the absence of explicit approach-avoidance labels, we had a considerable amount of participants that did not understand and correctly perform the requested action. Including those participants would have excessively increased variance in the analyses. **(2)** We did not exclude participants who reported cognitive troubles as the item did not prove able to detect effective troubles and this exclusion did not change the pattern of obtained results. **(3)** We did not exclude participants according to their cybersickness score as there is no clear exclusion criterion for cybersickness in the literature and the Simulator Sickness Questionnaire (Kennedy et al., 1993) is not built for cybersickness *per se* and is very sensitive (“merely closing one’s eyes for an extended period of time can affect the measurement,” Rebenitsch and Owen, 2016). Moreover, we exposed participants to VR for approximately 15 min which induces generally low levels of cybersickness (Stanney et al., 2002; Pan and Hamilton, 2018). Again, excluding those extreme participants (which were outliers on studentized residuals, that is above four, when running a simple regression analysis on cybersickness scores alone; Judd et al., 2011) did not change the pattern of results.

9 As experimenters have to insert manually the script in the file read by the application, they were not blind to conditions.

10 With four observations deleted due to missing values on task judgment. As pleasantness judgment, difficulty judgment, simulator sickness and presence did not differ between approach and avoidance conditions in both studies, we did not include them in the models. Moreover, unlike the four pilots, the difficulty, pleasantness and tiredness judgment of the task in this study were confounded with judgments of the VR due to item wording problems.

11 For the sake of clarity, we reported the antilog of log-transformed means.

12 We excluded one participant due to missing data on the Presence Questionnaire.

13 Again we considered only data of the first bloc for Pilot 1.

## References

[B1] Alexopoulos T. Ric F. (2007). The evaluation-behavior link: direct and beyond valence. *J. Exp. Soc. Psychol.* 43 1010–1016. 10.1016/j.jesp.2006.10.017

[B2] Bailenson J. N. Blascovich J. Beall A. C. Loomis J. M. (2003). Interpersonal distance in immersive virtual environments. *Pers. Soc. Psychol. Bull.* 29 819–833. 10.1177/0146167203029007002 15018671

[B3] Barsalou L. W. (1999). Perceptions of perceptual symbols. *Behav. Brain Sci.* 22 637–660. 10.1017/S0140525X99532147 11301525

[B4] Barsalou L. W. (2003). Situated simulation in the human conceptual system. *Lang. Cogn. Process.* 18 513–562. 10.1111/j.1551-6709.2010.01168.x 21564270

[B5] Barsalou L. W. (2005). “Situated conceptualization,” in *Handbook of Categorization in Cognitive Science*, eds Cohen H. Lefebvre C. (Amsterdam: Elsevier), 619–650. 10.1016/b978-008044612-7/50083-4

[B6] Barsalou L. W. (2008). Grounded cognition. *Annu. Rev. Psychol.* 59 617–645. 10.1146/annurev.psych.59.103006.093639 17705682

[B7] Barsalou L. W. (2015). “Situated conceptualization: theory and applications,” in *Foundations of Embodied Cognition, Volume 1: Perceptual and Emotional Embodiment*, eds Coello Y. Fischer M. H. (East Sussex: Psychology Press), 11–37.

[B8] Beatty G. F. Cranley N. M. Carnaby G. Janelle C. M. (2016). Emotions predictably modify response times in the initiation of human motor actions: a meta-analytic review. *Emotion* 16 237–251. 10.1037/emo0000115 26461243

[B9] Blascovich J. Loomis J. Beall A. C. Swinth K. R. Hoyt C. L. Bailenson J. N. (2002). Immersive virtual environment technology as a methodological tool for social psychology. *Psychol. Inq.* 13 103–124. 10.1207/S15327965PLI1302_01

[B10] Bouchard S. Robillard G. Renaud P. (2007). Revising the factor structure of the simulator sickness questionnaire. *Annu. Rev. Cyberther. Telemed.* 5 128–137.

[B11] Bouman D. Stins J. F. (2018). Back off! the effect of emotion on backward step initiation. *Hum. Mov. Sci.* 57 280–290. 10.1016/j.humov.2017.09.006 28919167

[B12] Bower G. H. (1981). Mood and memory. *Am. Psychol.* 36 129–148. 7224324 10.1037//0003-066x.36.2.129

[B13] Cacioppo J. T. Priester J. R. Berntson G. G. (1993). Rudimentary determinants of attitudes: II. Arm flexion and extension have differential effects on attitudes. *J. Personal. Soc. Psychol.* 65 5–17. 10.1037/0022-3514.65.1.5 8355142

[B14] Carassa A. Morganti F. Tirassa M. (2005). “A situated cognition perspective on presence,” in *Proceedings of the 27th Annual Meeting of the Cognitive Science Society*, (Mahwah, NJ: Erlbaum).

[B15] Carver C. S. Scheier M. F. (2000). “On the structure of behavioral self-regulation,” in *Handbook of Self-Regulation*, eds Boekaerts M. Pintrich P. R. Zeidner M. (Cambridge, MA: Academic Press), 41–84. 10.1016/b978-012109890-2/50032-9

[B16] Centerbar D. B. Clore G. L. (2006). Do approach-avoidance actions create attitudes? *Psychol. Sci.* 17 22–29. 10.1111/j.1467-9280.2005.01660.x 16371140

[B17] Cesario J. Plaks J. E. Hagiwara N. Navarrete C. D. Higgins E. T. (2010). The ecology of automaticity: how situational contingencies shape action semantics and social behavior. *Psychol. Sci.* 21 1311–1317. 10.1177/0956797610378685 20660891

[B18] Chen M. Bargh J. A. (1999). Consequences of automatic evaluation: immediate behavioral predispositions to approach or avoid the stimulus. *Personal. Soc. Psychol. Bull.* 25 215–224. 10.1177/0146167299025002007

[B19] Chen S. Ybarra O. Kiefer A. K. (2004). Power and impression formation: the effects of power on the desire for morality and competence information. *Soc. Cogn.* 22 391–421. 10.1521/soco.22.4.391.38296

[B20] Corr P. J. Cooper A. J. (2016). The reinforcement sensitivity theory of personality questionnaire (RST-PQ): development and validation. *Psychol. Assess.* 28 1427–1440. 10.1037/pas0000273 26845224

[B21] Corr P. J. McNaughton N. (2012). Neuroscience and approach/avoidance personality traits: a two stage (valuation–motivation) approach. *Neurosci. Biobehav. Rev.* 36 2339–2354. 10.1016/j.neubiorev.2012.09.013 23041073

[B22] Damasio A. R. (1989). Time-locked multiregional retroactivation: a systems-level proposal for the neural substrates of recall and recognition. *Cognition* 33 25–62. 10.1016/0010-0277(89)90005-x 2691184

[B23] De Houwer J. Crombez G. Baeyens F. Hermans D. (2001). On the generality of the affective Simon effect. *Cogn. Emot.* 15 189–206. 10.1080/0269993004200051

[B24] Dotsch R. Wigboldus D. H. (2008). Virtual prejudice. *J. Exp. Soc. Psychol.* 44 1194–1198. 10.1016/j.jesp.2008.03.003

[B25] Dru V. Cretenet J. (2008). Influence of unilateral motor behaviors on the judgment of valenced stimuli. *Cortex* 44 717–727. 10.1016/j.cortex.2006.11.004 18472041

[B26] Elliot A. J. Covington M. V. (2001). Approach and avoidance motivation. *Educ. Psychol. Rev.* 13 73–92.

[B27] Fayant M. P. Muller D. Nurra C. Alexopoulos T. Palluel-Germain R. (2011). Moving forward is not only a metaphor: approach and avoidance lead to self-evaluative assimilation and contrast. *J. Exp. Soc. Psychol.* 47 241–245. 10.1016/j.jesp.2010.07.013

[B28] Förster J. Liberman N. Friedman R. S. (2007). Seven principles of goal activation: a systematic approach to distinguishing goal priming from priming of non-goal constructs. *Pers. Soc. Psychol. Rev.* 11 211–233. 10.1177/1088868307303029 18453462

[B29] Galton F. (1884). Measurement of character. *Fortn. Rev.* 42 179–185.

[B30] Gast A. Gawronski B. De Houwer J. (2012). Evaluative condi-tioning: recent developments and future directions. *Learn. Motiv.* 43 79–88. 10.1016/j.lmot.2012.06.004

[B31] Institut National de la Statistique et des Etudes Economiques [INSEE] (2015). *Census of First Names given to Children Born in France from 1990 to 2015.* Available at: https://www.insee.fr/fr/statistiques/2540004 (accessed November 29, 2017).

[B32] James W. (1904). Does “consciousness” exist? *J. Philos. Psychol. Sci. Methods* 1 477–491. 10.2307/2011942

[B33] Jones C. R. Vilensky M. R. Vasey M. W. Fazio R. H. (2013). Approach behavior can mitigate predominately univalent negative attitudes: evidence regarding insects and spiders. *Emotion* 13 989–996. 10.1037/a0033164 23795593

[B34] Judd C. M. McClelland G. H. Ryan C. S. (2011). *Data Analysis: A Model Comparison Approach.* Abingdon: Routledge

[B35] Kawakami K. Phills C. E. Steele J. R. Dovidio J. F. (2007). (Close) distance makes the heart grow fonder: improving implicit racial attitudes and interracial interactions through approach behaviors. *J. Pers. Soc. Psychol.* 92 957–971. 10.1037/0022-3514.92.6.957 17547482

[B36] Kennedy R. S. Lane N. E. Berbaum K. S. Lilienthal M. G. (1993). Simulator sickness questionnaire: an enhanced method for quantifying simulator sickness. *Int. J. Aviat. Psychol.* 3 203–220. 10.1207/s15327108ijap0303_3

[B37] Kim R. S. (2011). *Standardised Regression Coefficients as Indices of Effect Sizes in Meta-Analysis.* PhD Dissertation The Florida State University College of Education: Tallahassee, FL.

[B38] Koch S. Holland R. W. Hengstler M. van Knippenberg A. (2009). Body locomotion as regulatory process: stepping backward enhances cognitive control. *Psychol. Sci.* 20 549–550. 10.1111/j.1467-9280.2009.02342.x 19476588

[B39] Krieglmeyer R. De Houwer J. Deutsch R. (2011). How farsighted are behavioral tendencies of approach and avoidance? The effect of stimulus valence on immediate vs. ultimate distance change. *J. Exp. Soc. Psychol.* 47 622–627. 10.1016/j.jesp.2010.12.021

[B40] Krieglmeyer R. Deutsch R. (2010). Comparing measures of approach-avoidance behaviour: the manikin task vs. two versions of the joystick task. *Cogn. Emot.* 24 810–828. 10.1080/02699930903047298

[B41] Krishna A. Eder A. B. (2019). The influence of pre-training evaluative responses on approach-avoidance training outcomes. *Cogn. Emot.* 10.1080/02699931.2019.1568230. [Epub ahead of print]. 30663944

[B42] Labeye E. Versace R. (2007). “Activation and integration of sensory component,” in *Proceedings of the XVth Conference of the European Society for Cognitive Psychology (ESCOP)*, eds Grainger J. Alario F. X. Burle B. Janssen N. (Marseille).

[B43] Laham S. M. Kashima Y. Dix J. Wheeler M. Levis B. (2014). Elaborated contextual framing is necessary for action-based attitude acquisition. *Cogn. Emot.* 28 1119–1126. 10.1080/02699931.2013.867833 24354687

[B44] Loomis J. M. (1992). Distal attribution and presence. *Presence (Camb).* 1 113–118. 10.1162/pres.1992.1.1.113

[B45] Ma D. S. Correll J. Wittenbrink B. (2015). The Chicago face database: a free stimulus set of faces and norming data. *Behav. Res. Methods* 47 1122–1135. 10.3758/s13428-014-0532-5 25582810

[B46] Markman A. B. Brendl C. M. (2005). Constraining theories of embodied cognition. *Psychol. Sci.* 16 6–10. 10.1111/j.0956-7976.2005.00772.x 15660844

[B47] McCall C. (2015). “Mapping social interactions: the science of proxemics,” in *Social Behavior from Rodents to Humans. Current Topics in Behavioral Neurosciences*, Vol. 30 eds Wöhr M. Krach S. (Berlin: Springer), 295–308. 10.1007/7854_2015_431 26728171

[B48] McNaughton N. DeYoung C. Corr P. J. (2016). “Approach and avoidance,” in *Neuroimaging Personality and Character: Traits and Mental States in the Brain*, eds Absher J. R. Cloutier J. (Amsterdam: Elsevier), 25–49. 10.1016/B978-0-12-800935-2.00002-6

[B49] Mehrabian A. (1968). Relationship of attitude to seated posture, orientation, and distance. *J. Pers. Soc. Psychol.* 10 26–30. 10.1037/h0026384 5682520

[B50] Mennecke B. E. Triplett J. L. Hassall L. M. Conde Z. J. Heer R. (2011). An examination of a theory of embodied social presence in virtual worlds. *Decis. Sci.* 42 413–450. 10.1111/j.1540-5915.2011.00317.x

[B51] Merleau-Ponty M. (1964). “Eye and mind,” in *The Primacy of Perception and Other Essays*, ed. James Edie M. (Evanston, IL: Northwestern University Press).

[B52] Mertens G. Van Dessel P. De Houwer J. (2018). The contextual malleability of approach-avoidance training effects: approaching or avoiding fear conditioned stimuli modulates effects of approach-avoidance training. *Cogn. Emot.* 32 341–349. 10.1080/02699931.2017.1308315 28345433

[B53] Niedenthal P. M. Barsalou L. W. Winkielman P. Krauth-Gruber S. Ric F. (2005). Embodiment in attitudes, social perception, and emotion. *Pers. Soc. Psychol. Rev.* 9 184–211. 10.1207/s15327957pspr0903_1 16083360

[B54] Neumann R. Hülsenbeck K. Seibt B. (2004). Attitudes towards people with AIDS and avoidance behavior: automatic and reflective bases of behavior. *J. Exp. Soc. Psychol.* 40 543–550. 10.1016/j.jesp.2003.10.006

[B55] Oosterhof N. N. Todorov A. (2008). The functional basis of face evaluation. *Proc. Natl. Acad. Sci. U.S.A.* 105 11087–11092. 10.1073/pnas.0805664105 18685089 PMC2516255

[B56] Paladino M.-P. Castelli L. (2008). On the immediate consequences of intergroup categorization: activation of approach and avoidance motor behavior toward ingroup and outgroup members. *Personal. Soc. Psychol. Bull.* 34 755–768. 10.1177/0146167208315155 18388255

[B57] Pan X. Hamilton A. F. D. C. (2018). Why and how to use virtual reality to study human social interaction: the challenges of exploring a new research landscape. *Br. J. Psychol.* 109 395–417. 10.1111/bjop.12290 29504117 PMC6055846

[B58] Papies E. K. Barsalou L. W. (2015). “Grounding desire and motivated behavior: a theoretical framework and review of empirical evidence,” in *The Psychology of Desire*, eds Hofmann W. Nordgren L. F. (New York, NY: The Guilford Press), 36–60.

[B59] Parsons T. D. Rizzo A. A. (2008). Affective outcomes of virtual reality exposure therapy for anxiety and specific phobias: a meta-analysis. *J. Behav. Ther. Exp. Psychiatry* 39 250–261. 10.1016/j.jbtep.2007.07.007 17720136

[B60] Pecher D. Zwaan R. A. (2005). *Grounding Cognition: The Role of Perception and Action in Memory, Language, and Thinking.* Cambridge: Cambridge University Press.

[B61] Price T. F. Harmon-Jones E. (2010). The effect of embodied emotive states on cognitive categorization. *Emotion* 10 934–938. 10.1037/a0019809 21171763

[B62] Price T. F. Harmon-Jones E. (2016). “Embodying approach motivation: a review of recent evidence,” in *Advances in Motivation Science*, Vol. 3 ed. Elliot A. J. (Amsterdam: Elsevier Science), 81–111. 10.1016/bs.adms.2015.12.002

[B63] Rakover S. S. (1981). Social psychological theory and falsification. *Personal. Soc. Psychol. Bull.* 7 123–130. 10.1037/amp0000146 29345488 PMC5776753

[B64] Rebenitsch L. Owen C. (2016). Review on cybersickness in applications and visual displays. *Virtual Real.* 20 101–125. 10.1007/s10055-016-0285-9

[B65] Rinck M. Becker E. S. (2007). Approach and avoidance in fear of spiders. *J. Behav. Ther. Exp. Psychiatry* 38 105–120. 10.1016/j.jbtep.2006.10.001 17126289

[B66] Riskind J. H. (1984). They stoop to conquer: guiding and self-regulatory functions of physical posture after success and failure. *J. Pers. Soc. Psychol.* 47 479–493. 10.1037/0022-3514.47.3.479

[B67] Ritz T. Thöns M. (2006). Affective modulation of swallowing rates: unpleasantness or arousal? *J. Psychosom. Res.* 61 829–833. 10.1016/j.jpsychores.2006.05.008 17141673

[B68] Riva G. (2009). Is presence a technology issue? Some insights from cognitive sciences. *Virtual Real.* 13 159–169. 10.1007/s10055-009-0121-6

[B69] Riva G. Waterworth J. A. (2014). “Being present in a virtual world,” in *The Oxford Handbook of Virtuality*, ed. Grimshaw M. (New York, NY: Oxford University Press), 205–221.

[B70] Riva G. Waterworth J. A. Waterworth E. L. (2004). The layers of presence: a bio-cultural approach to understanding presence in natural and mediated environments. *Cyber Psychol. Behav.* 7 402–416. 10.1089/cpb.2004.7.402 15331027

[B71] Riva G. Waterworth J. A. Waterworth E. L. Mantovani F. (2011). From intention to action: the role of presence. *New Ideas Psychol.* 29 24–37. 10.1016/j.newideapsych.2009.11.002

[B72] Robillard G. Bouchard S. Renaud P. Cournoyer L. G. (2002). “Validation canadienne-française de deux mesures importantes en réalité virtuelle: l’Immersive tendencies questionnaire et le presence questionnaire,” in *Poster Presented at the 25th Meeting of the Société Québécoise Pour la Recherche en Psychologie (SQRP)*, (Trois-Rivières).

[B73] Rougier M. Muller D. Ric F. Alexopoulos T. Batailler C. Smeding A. (2018). A new look at sensorimotor aspects in approach/avoidance tendencies: the role of visual whole-body movement information. *J. Exp. Soc. Psychol.* 76 42–53. 10.1016/j.jesp.2017.12.004

[B74] Ruggiero G. Frassinetti F. Coello Y. Rapuano M. Di Cola A. S. Iachini T. (2017). The effect of facial expressions on peripersonal and interpersonal spaces. *Psychol. Res.* 81 1232–1240. 10.1007/s00426-016-0806-x 27785567

[B75] Schneirla T. (1959). “An evolutionary and developmental theory of biphasic processes underlying approach and withdrawal,” in *Nebraska Symposium on Motivation*, ed. Jones M. R. (Oxford: University of Nebraska Press), 1–42.

[B76] Seibt B. Neumann R. Nussinson R. Strack F. (2008). Movement direction or change in distance? Self- and object-related approach-avoidance motions. *J. Exp. Soc. Psychol.* 44 713–720. 10.1016/j.jesp.2007.04.013

[B77] Simmons J. P. Nelson L. D. Simonsohn U. (2013). “Life after p-hacking,” in *Proceedings of the Meeting of the Society for Personality and Social Psychology*, (New Orleans, LA), 17–19.

[B78] Slepian M. L. Young S. G. Rule N. O. Weisbuch M. Ambady N. (2012). Embodied impression formation: social judgments and motor cues to approach and avoidance. *Soc. Cogn.* 30 232–240. 10.1521/soco.2012.30.2.232

[B79] Smith P. K. Bargh J. A. (2008). Nonconscious effects of power on basic approach and avoidance tendencies. *Soc. Cogn.* 26 1–24. 10.1521/soco.2008.26.1.1 18568085 PMC2435045

[B80] Stanney K. M. Kingdon K. S. Graeber D. Kennedy R. S. (2002). Human performance in immersive virtual environments: effects of exposure duration, user control, and scene complexity. *Hum. Perform.* 15 339–366. 10.1207/s15327043hup1504_03

[B81] Stins J. F. Roelofs K. Villan J. Kooijman K. Hagenaars M. A. Beek P. J. (2011). Walk to me when I smile, step back when I’m angry: emotional faces modulate whole-body approach–avoidance behaviors. *Exp. Brain Res.* 212 603–611. 10.1007/s00221-011-2767-z 21698468 PMC3133774

[B82] Streicher M. C. Estes Z. (2016). Shopping to and fro: ideomotor compatibility of arm posture and product choice. *J. Consum. Psychol.* 26 325–336. 10.1016/j.jcps.2015.12.001

[B83] Topolinski S. Maschmann I. T. Pecher D. Winkielman P. (2014). Oral approach–avoidance: affective consequences of muscular articulation dynamics. *J. Pers. Soc. Psychol.* 106 885–896. 10.1037/a0036477 24841094

[B84] Van Dantzig S. Pecher D. Zwaan R. A. (2008). Approach and avoidance as action effects. *Q. J. Exp. Psychol.* 61 1298–1306. 10.1080/17470210802027987 19086189

[B85] Van Dessel P. De Houwer J. Gast A. Smith C. T. (2015). Instruction-Based approach-avoidance effects: changing stimulus evaluation via the mere instruction to approach or avoid stimuli. *Exp. Psychol.* 62 161–169. 10.1027/1618-3169/a000282 25516008

[B86] Van Dessel P. Eder A. B. Hughes S. (2018a). Mechanisms underlying effects of approach-avoidance training on stimulus evaluation. *J. Exp. Psychol. Learn. Mem. Cogn.* 44 1224–1241. 10.1037/xlm0000514 29648864

[B87] Van Dessel P. Hughes S. De Houwer J. (2018b). How do actions influence attitudes? An inferential account of the impact of action performance on stimulus evaluation. *Personal. Soc. Psychol. Rev.* 10.1177/1088868318795730. [Epub ahead of print]. 30229697

[B88] Varela F. J. Thompson E. Rosch E. (1991). *The Embodied Mind.* Cambridge, MA: MIT Press.

[B89] Versace R. Vallet G. T. Riou B. Lesourd M. Labeye É. Brunel L. (2014). Act-In: an integrated view of memory mechanisms. *J. Cogn. Psychol.* 26 280–306. 10.1080/20445911.2014.892113 29019445

[B90] Wentura D. Rothermund K. Bak P. (2000). Automatic vigilance: the attention-grabbing power of approach-and avoidance-related social information. *J. Pers. Soc. Psychol.* 78 1024–1037. 10.1037/0022-3514.78.6.1024 10870906

[B91] Wiers R. W. Eberl C. Rinck M. Becker E. S. Lindenmeyer J. (2011). Retraining automatic action tendencies changes alcoholic patients’ approach bias for alcohol and improves treatment outcome. *Psychol. Sci.* 22 490–497. 10.1177/0956797611400615 21389338

[B92] Willans T. Rivers S. Prasolova-Førland E. (2015). “Enactive emotion and presence in virtual environments,” in *Emotions, Technology, and Behaviors*, eds Tettegah S. Y. Espelage D. L. (London: Academic Press), 181–210. 10.1016/b978-0-12-801873-6.00010-8

[B93] Wilson M. (2002). Six views of embodied cognition. *Psychon. Bull. Rev.* 9 625–636. 10.3758/bf03196322 12613670

[B94] Witmer B. G. Singer M. J. (1998). Measuring presence in virtual environments: a presence questionnaire. *Presence* 7 225–240. 10.1162/105474698565686

[B95] Word C. O. Zanna M. P. Cooper J. (1974). The nonverbal mediation of self-fulfilling prophecies in interracial interaction. *J. Exp. Soc. Psychol.* 10 109–120. 10.1016/0022-1031(74)90059-6

[B96] Worthington M. E. (1974). Personal space as a function of the stigma effect. *Environ. Behav.* 6 289–294. 10.1177/001391657400600302

[B97] Woud M. L. Becker E. S. Lange W. G. Rinck M. (2013a). Effects of approach-avoidance training on implicit and explicit evaluations of neutral, angry, and smiling face stimuli. *Psychol. Rep.* 113 199–216. 10.2466/21.07.pr0.113x10z1 24340811

[B98] Woud M. L. Maas J. Becker E. S. Rinck M. (2013b). Make the manikin move: symbolic approach-avoidance responses affect implicit and explicit face evaluations. *J. Cogn. Psychol.* 25 738–744. 10.1080/20445911.2013.817413

[B99] Yzerbyt V. Y. Leyens J. P. Corneille O. (1998). Social judgeability and the bogus pipeline: the role of naive theories of judgment in impression formation. *Soc. Cogn.* 16 56–77. 10.1521/soco.1998.16.1.56

