# Supplementary material for: “Science Manipulates the Things and Lives in Them”: Reconsidering Approach-Avoidance Operationalization Through a Grounded Cognition Perspective
Source: Front Psychol. 2019 Jun 25;10:1418. doi: 10.3389/fpsyg.2019.01418 (PMC6603219; doi:10.3389/fpsyg.2019.01418)
Supplement: Supplementary file 1 [file Data_Sheet_1.pdf]

Supplementary Material 1. Pilots details

We reported only the effects we are interested in, but analysis outputs were given at the end of this file.

1 Pilot 1

1.1 Method

1.1.1 Participants

Fifty psychology undergraduate students participated in the study on the voluntary basis or for course credits.

1.1.2 Material

Sixty computerized faces (30 men and 30 women faces) were selected from the Randomly Generated Faces Database ($M_{Attractiveness}$= 4.30, $SE_{Attractiveness}$ = 0.01, from a scale ranging from 1: *not at all* to 9: *extremely*, Oosterhof &Todorov, 2008) so as to be as neutral in positivity as possible.

1.1.3 Procedure

Participants were seated at a computer between two wooden boards on each one of which we fixed a computer mouse (see Figure 1). They were instructed that as part of an ergonomic study in social network they will have to greet individuals while performing different movements. We informed them that in one block, they will have to lean their chest forward and while in the other block they will have to lean their chest backward. Each trial began with a cross fixation during 500 ms followed by an individual's face. After 750 ms, a speech bubble "hello" appeared on the screen, indicating that the individual greeted the participant. In the first block, half participants had to lean their chest forward while the other half had to lean their chest backward in order to reach the corresponding computer mouse (behind vs. in front of them). The mouse click made a response bubble "hello" appeared on the screen indicating that the participant responded to the individual. Then, participants had to return to the central position (i.e., straight back) and press the "enter" key on the keyboard they had in their hands. After what, they indicated to what extent they judge the face pleasant from 1: *very unpleasant* to 7: *very pleasant*. The next trial began after 200 ms. In the second block, chest inclination was reversed. Across participants, block order was counterbalanced.

Finally, participants indicated to what extent they found the task in each block pleasant, difficult and tiring from 0: *not at all* to 6: *very much*, probed for suspicions and debriefed.

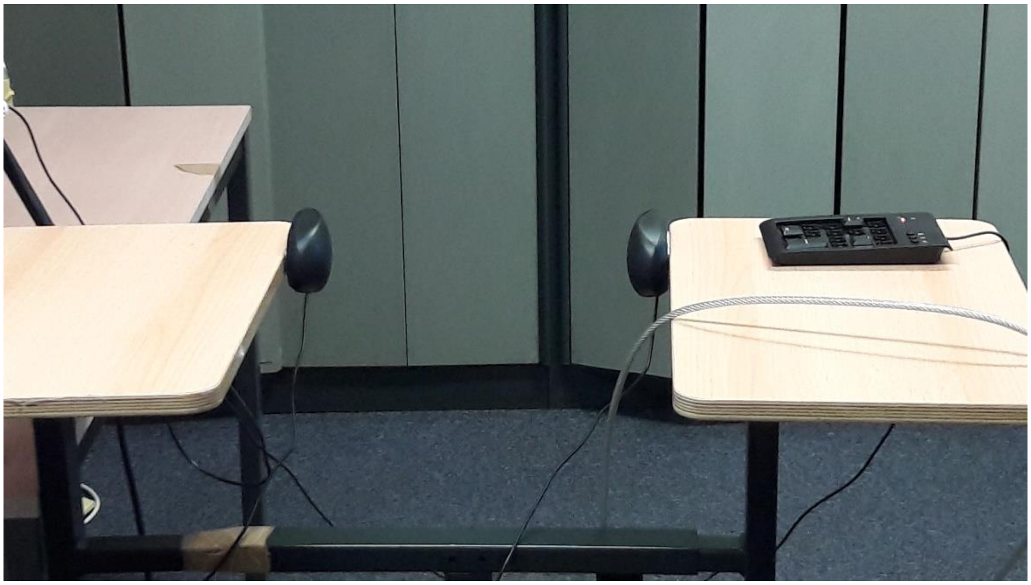

Figure 1. Two wooden-boards devices.

1.2 Results

We suppressed evaluation given after 5000 ms (2.8 %) as such evaluations were less likely to reflect spontaneous judgment[1].

We estimated a model with movement (approach, avoidance), order (approach first, avoidance first), pleasantness, difficulty and tiredness of the task as fixed factors and participants and stimuli as random factors. The main effect of movement was significant with more positive evaluations in the approach (M = 3.78, SE = 0.16) than in the avoidance condition (M = 3.54, SE = 0.15), F (1, 2333) = 9.11, p = .0026, β = 0.16, 95 % CI [0.05, 0.26]. However this effect was qualified by a movement by order interaction, F (1, 2333) = 11.42, p < .0001, β = -0.36, 95 % CI [-0.56, -0.15]. Participants performing approach first had better evaluation of individuals when approaching them (M = 4.00, SE = 0.24) than when avoiding them (M = 3.51, SE = 0.20), F (1, 2212) = 13.26, p < .0001, β = 0.34, 95 % CI [0.15, 0.52]. However, this was not the case for those performing avoidance first, F < 1, p = .71, β = -0.02, 95 % CI [-0.12, 0.08], (M_{Approach} = 3.54, SE_{Approach} = 0.20; $M_{\text{Avoidance}}$ = 3.57, $SE_{\text{Avoidance}}$ = 0.21).

Due to this movement by order interaction, we decided to focus our attention on the first block less susceptible to be impacted by experimental demand and tiredness effects. We estimated the same model than previously except that we did not include order as a fixed factor. The results revealed a non-significant and negative effect of approach on evaluations compared to avoidance, F (1, 33.98) < 1, p = .518, β = -0.18, 95 % CI [-0.65, 0.28], (M_{Approach} = 3.44, SE_{Approach} = 0.36; $M_{\text{Avoidance}}$ = 3.70, $SE_{\text{Avoidance}}$ = 0.21).

1.3 Discussion

In Pilot 1, we implemented a whole-body approach-avoidance manipulation in the study of their influence on evaluations. If the results shown more positive evaluations for approached than for

[1] Including these trials did not change the pattern of results.

avoided faces, this effect was mostly due to a decrease in evaluations when participant performed avoidance in the second block. This effect was not obtained when focusing on the first block and even tend in the opposite direction (but the first block analysis is necessarily underpowered). Moreover, participants made the movement opposite to the one manipulated when returning to the central position before evaluating individuals (e.g., approach after leaning the chest backward). Those movements may have produced some ambiguity in our whole-body manipulation of approach-avoidance. Finally, one could question the part of desirability bias in this pilot as we relied on a judgmental evaluation item. Therefore, we targeted these particular issues in a between-subject design second pilot.

2 Pilot 2

2.1 Method

2.1.1 Participants

In Pilot 2, 108 psychology undergraduate students participated in the study on the voluntary basis or for course credits. Upon their arrival, they were randomly assigned to the approach or the avoidance condition. We excluded one participant for having suspicion about our hypothesis.

2.1.2 Material

We relied on the same 60 computerized faces than in Pilot 1 that we presented with a chest and inserted in a neutral room background.

2.1.3 Procedure

Participants were introduced as takin part in a study on impression formation during job interview. They were installed between the same devices as in Pilot 1 (two wooden boards) but faced the wall rather than the computer. On the wall, we projected the stimuli in real size and placed a table in front of them to simulate a situation of interaction (see Figure 2). Each trial began with a 500 ms fixation cross followed by the contextualized individual’s face. In order to indicate that the individual greeted the participant, a speech bubble “hello” appeared on the screen after a random duration (from 500 ms to 1400 ms). Depending on the condition, participants had to say “hello” aloud while leaning their chest forward or backward in order to reach the computer mouse. After what, the evaluation item appeared on the screen and participants had to maintain their position while giving their impression about the individual (from -3: *I don’t like at all* to +3: *I like very much*). Participants were instructed to respond quickly and spontaneously. Then, they returned to the central position and pressed the “enter” to finish the trial. If participants responded before the speech bubble appeared, they received a warning feedback and the trial ended after 1500 ms.

At the end of the task, participants indicated to what extent they found it pleasant, difficult and tiring participants were finally for suspicions and debriefed.

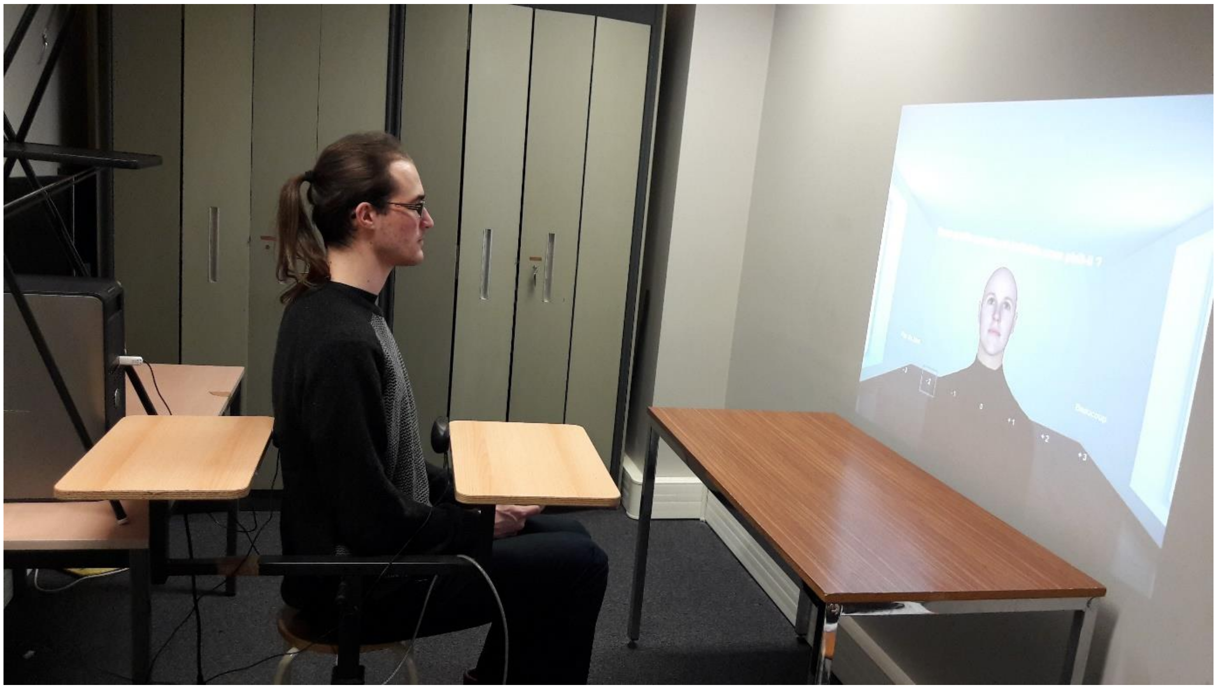

Figure 2. Pilot 2 configuration.

2.2 Results

We suppressed evaluation given after 5000 ms (11.11 %)[2] and trials for which participants responded before the speech bubble apparition (1.5 %).

We estimated a model with movement (approach, avoidance), pleasantness, difficulty and tiredness of the task as fixed factors and participants and stimuli as random factors. We obtained a marginal main effect of movement opposite to the one expected with more negative evaluations in the approach (M = 0.11, SE = 0.12) than in the avoidance condition (M = 0.35, SE = 0.12), F (1, 90.15) = 2.81, p = .097, β = -0.18, 95 % CI [-0.36, 0.01]. This effect was qualified by a three-way interaction between movement, difficulty and pleasantness, F (1, 90.70) = 4.44, p = .038, β = -0.09, 95 % CI [-0.17, -0.01].

2.3 Discussion

In Pilot 2, while we increased the ecological nature of the situation by mean of contextualization, we failed to obtain the positive influence of approach on evaluations compared to avoidance. Even, participants evaluated more negatively individuals they approached than those they avoided. However, the job interview cover-story coupled with our two wooden-boards devices may have changed the meaning of avoidance in terms of expansion (or even power) and the meaning of approach in terms of introversion (or even submission). We are not aware to what extent this could have influence the results but we remedied to this eventuality in Pilot 3. We also relied on an approach-avoidance operationalization, already used in previous research: chest postures (Price & Harmon-Jones, 2010).

[2] Including these trials did not change the pattern of results.

3 Pilot 3

3.1 Method

3.1.1 Participants

In Pilot 3, 101 psychology undergraduate students participated in the study on the voluntary basis and were randomly assigned to the approach or avoidance condition. We excluded participants for having some psychomotor troubles (1) or having suspicions about our main hypothesis (3). We thus analyzed the remaining 97 participants.

3.1.2 Procedure

Pretexting a study on impression formation, we asked participants to give their impression of individuals, while maintaining their chest leant forward or backward. Participants in the approach condition were instructed to lean their chest until their shoulders align with two stickers placed on the front edge of the chair and press the palm of their hand in the back edge of the chair. Participants in the avoidance condition were instructed to push the backrest of the chair with their back and put their hand on the stickers placed on the front edge of the chair.

We presented stimuli on a computer screen and reminded participants to maintain the correct position every 20 presented individuals. Each trial began with a fixation cross during 500 ms followed by the individual. After 1500 ms the evaluation item appeared on the screen and participants gave their impression about the individual orally (from -3: *I don't like at all* to +3: *I like very much*). After what the experimenter manually ended the trial.

At the end of the 60 trials, participants indicated to what extent they found the task pleasant, difficult and tiring, were probed for suspicions and debriefed.

3.2 Results

We estimated a model with posture (approach, avoidance), pleasantness, difficulty and tiredness of the task as fixed factors and participants and stimuli as random factors. We did not obtain the expected main effect of posture, F (1, 81) = 0.62, p = .434, β = -0.08, 95 % CI [-0.26, 0.10]. Descriptively, participants had more negative evaluations in the approach (M = 0.13, SE = 0.20) than in the avoidance condition (M = 0.23, SE = 0.19).

3.3 Discussion

Although relying on an approach-avoidance operationalization already used in the literature, we failed to obtain the positive influence of approach (compared to avoidance) on evaluations in Pilot 3. Again, evaluations were descriptively in the opposite direction with more negative evaluation in the approach than in the avoidance condition. As we dealt with interpersonal evaluations it was possible that participants did not feel allowed to use their feeling in order to evaluate individuals. We tried to circumvent this eventuality in Pilot 4 by making participants believe they received pseudo-individualizing information about each presented individuals in a subliminal way (but in fact, we did not). With such believe, participants should feel more entitled to evaluate the individuals in line with their feeling (Yzerbyt, Leyens, & Corneille, 1998).

4 Pilot 4

4.1 Method

4.1.1 Participants

One hundred fifty six psychology undergraduate students participated in the study on the voluntary basis and were partially randomly assigned to the approach, avoidance or control condition[3]. We excluded participant who understood the aim of the study and one participant with prosopagnosia. We thus analyzed the data of the remaining 154 participants.

4.1.2 Material

From the Chicago Face Database (Ma et al., 2015) we selected pictures of 15 men and 15 women ($M_{Attractiveness}$ = 3.37, $SE_{Attractiveness}$ = 0.12; on a scale ranging from 1: *not at all* to 7: *extremelly*).

4.1.3 Procedure

Participants were instructed to give their impression of individuals orally, while maintaining their chest leant forward, backward or upright. Participants in the approach and the avoidance conditions received the same instructions as in Pilot 3. Participants in the control condition were instructed to maintain their chest upright and press the palm of their hand on both sides of the chair.

We presented stimuli on a computer screen and reminded participants to maintain the correct position every 20 presented individuals. We also explain to participants that information about each individuals would be presented in a subliminal way before they saw their faces (e.g., their names, their hobbies, etc.). Thus, after a 500 ms fixation cross, we presented a noise picture during 30 ms before the individual's picture. Importantly none information was presented throughout the noise picture duration. After 1500 ms the evaluation item appeared on the picture and participants gave their impression about the individual orally (from -3: *I don't like at all* to +3: *I like very much*). As in Pilot 3, the experimenter manually ended the trial.

Finally, participants indicated to what extent they found the task pleasant, difficult and tiring, were probed for suspicions and debriefed.

4.2 Results

We created two codes of contrast in order to test the linear effect of posture. In the first contrast, we opposed the approach (1) to the avoidance condition (-1) ignoring the control condition (0). In the second contrast, we opposed the control condition (2) with approach (-1) and avoidance conditions (-1). We estimated a model with the codes of contrasts for posture, pleasantness, difficulty and tiredness of the task as fixed factors and participants and stimuli as random factors. The first contrast did reveal that evaluations were not significantly more positive in the approach (M = 0.56, SE = 0.14) than in the avoidance condition (M = 0.45, SE = 0.13), F (1, 130) = 0.70, p = .406, β_Z = 0.04, 95% IC [-0.04, 0.12]. The control condition did not fall between the approach and avoidance ones, F (1, 130) = 1.10, p = .30, β_Z = -0.02, 95% IC [-0.07, 0.02], (M = 0.40, SE = 0.13).

4.3 Discussion

In Pilot 4, while relying on whole-body approach-avoidance operationalization that have been previously used in the literature, we failed to show their effect on evaluations. However, the results

[3] We decided to include the control condition after the study began. Data from 66 participants were already collected.

are in the expected direction with more positive evaluations in the approach than in the avoidance condition.

5 Mini-meta analysis

In order to see the forest for the trees, we ran a random effects mini meta-analysis (with the "metafor" R package) on the standardized regression coefficients (Kim, 2011). This meta-analysis revealed a statistically non-significant effect of approach-avoidance behaviors on evaluations, $z = -0.80$, $p = .424$, $\beta_Z = -0.05$, 95% CI [-0.17, 0.07][4]. The overall effect size even points to the opposite direction of our hypothesis: participants tend to evaluate individuals more negatively when they approach than when they avoid them.

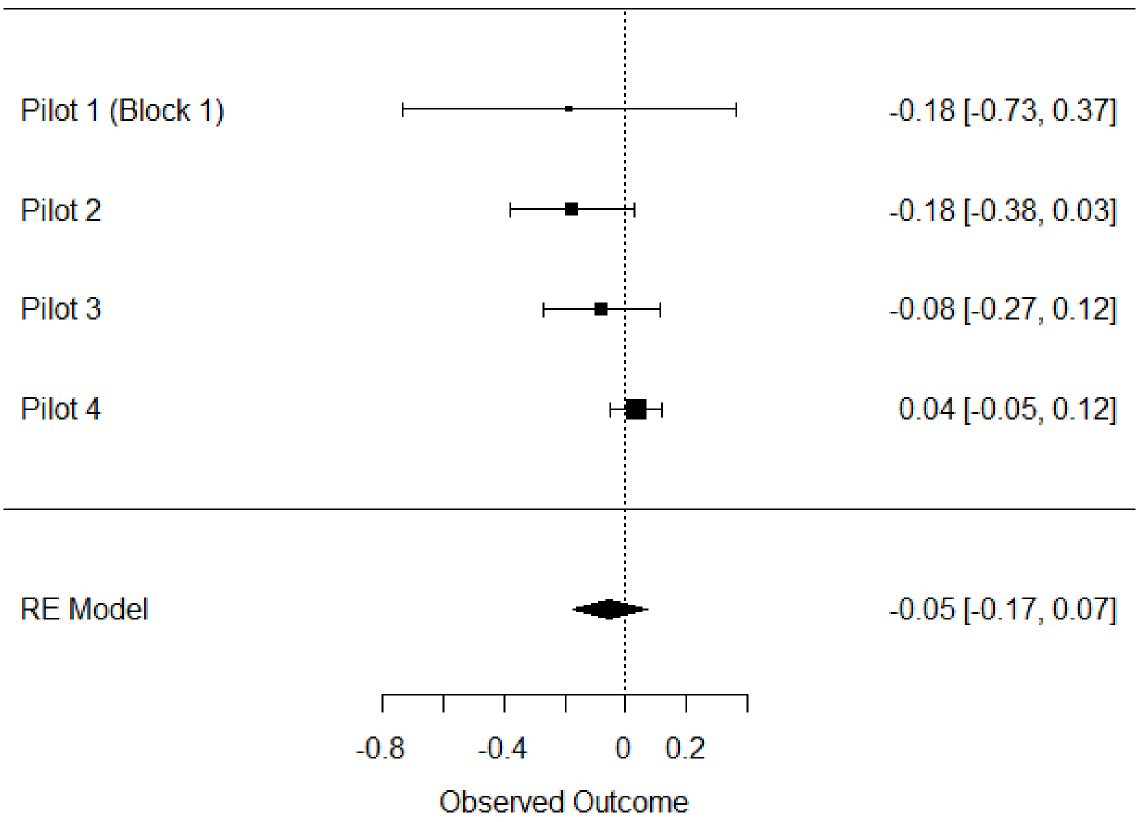

Figure 3. Meta-analysis of the four pilots. At the left are reported the standardized regression coefficients and their confidence intervals.

[4] We only considered the first bloc for Pilot 1 in the meta-analysis. Even if by doing that we lack power, this was done for comparability purpose with the other pilots that were in a between subject design and because we obtained an interaction between movement and bloc order.
