# Supplementary material for: “Science Manipulates the Things and Lives in Them”: Reconsidering Approach-Avoidance Operationalization Through a Grounded Cognition Perspective
Source: Front Psychol. 2019 Jun 25;10:1418. doi: 10.3389/fpsyg.2019.01418 (PMC6603219; doi:10.3389/fpsyg.2019.01418)
Supplement: Supplementary file 2 [file Data_Sheet_2.pdf]

Supplementary Material 2. R Outputs analysis for Study 1 and Study 2

1 Study 1

1.1 Evaluations.

```
Linear mixed model fit by REML
t-tests use  Satterthwaite approximations to degrees of freedom ['lmerMod']
Formula: EvalZ ~ Condition_C1 * Condition_C2 * Tiringc + (1 | pp) + (1 |      Perso)
   Data: EVAL_Mn

REML criterion at convergence: 10633.3

Scaled residuals:
    Min      1Q  Median      3Q     Max
-3.4841 -0.6393  0.0136  0.6733  3.9711

Random effects:
 Groups   Name        Variance Std.Dev.
 pp       (Intercept) 0.1785   0.4225
 Perso    (Intercept) 0.2681   0.5178
 Residual             0.5695   0.7546
Number of obs: 4450, groups:  pp, 158; Perso, 30

Fixed effects:
                       Estimate Std. Error         df t value Pr(>|t|)
(Intercept)           -0.013606   0.101363  37.100000  -0.134    0.894
Condition_C1          -0.028534   0.045788 153.090000  -0.623    0.534
Condition_C2           0.003363   0.025272 151.280000   0.133    0.894
Tiringc               -0.019006   0.022767 152.010000  -0.835    0.405
Condition_C1:Tiringc  -0.032743   0.027784 152.980000  -1.178    0.240
Condition_C2:Tiringc  -0.002863   0.016156 151.060000  -0.177    0.860
```

1.2 Action tendencies.

```
Linear mixed model fit by REML
t-tests use  Satterthwaite approximations to degrees of freedom ['lmerMod']
Formula: VAAST.RT.logZ ~ Condition_C1 * Condition_C2 * Movementc * Tiringc +      (1 | pp) + (1 | First_Name)
   Data: VAAST_Mn

REML criterion at convergence: 36558.6

Scaled residuals: 
    Min      1Q  Median      3Q     Max 
-4.1802 -0.6648 -0.1226  0.5335  4.3743 

Random effects:
 Groups     Name        Variance Std.Dev.
 pp         (Intercept) 0.30572  0.5529  
 First_Name (Intercept) 0.01311  0.1145  
 Residual               0.68855  0.8298  
Number of obs: 14545, groups:  pp, 158; First_Name, 24

Fixed effects:
                                 Estimate Std. Error         df t value Pr(>|t|)    
(Intercept)                    -9.087e-03  5.148e-02  1.580e+02  -0.176   0.8601    
Condition_C1                   -4.550e-02  5.735e-02  1.520e+02  -0.793   0.4288    
Condition_C2                    3.355e-02  3.175e-02  1.520e+02   1.057   0.2923    
Movementc                      -1.123e-01  1.417e-02  1.436e+04  -7.925 2.44e-15 ***
Tiringc                         4.109e-02  2.857e-02  1.520e+02   1.438   0.1524    
Condition_C1:Movementc          4.351e-03  1.772e-02  1.436e+04   0.245   0.8061    
Condition_C2:Movementc          2.642e-03  9.806e-03  1.436e+04   0.269   0.7876    
Condition_C1:Tiringc           -9.497e-03  3.481e-02  1.520e+02  -0.273   0.7853    
Condition_C2:Tiringc           -1.458e-02  2.030e-02  1.520e+02  -0.718   0.4738    
Movementc:Tiringc              -1.591e-02  8.834e-03  1.436e+04  -1.801   0.0717 .  
Condition_C1:Movementc:Tiringc -6.960e-03  1.078e-02  1.436e+04  -0.646   0.5185    
Condition_C2:Movementc:Tiringc -8.573e-03  6.269e-03  1.436e+04  -1.368   0.1715    
---
Signif. codes:  0 '***' 0.001 '**' 0.01 '*' 0.05 '.' 0.1 ' ' 1
```

1.3 Neuropsychological systems.

```
Call:
lm(formula = BASZ ~ Condition_C1 + Condition_C2 + Tiringc + Condition_C1:Tiringc + 
    Condition_C2:Tiringc, data = RSTPQ)

Residuals:
     Min       1Q   Median       3Q      Max 
-2.80471 -0.52511  0.07925  0.52345  2.19951 

Coefficients:
                     Estimate Std. Error t value Pr(>|t|)
(Intercept)          -0.04561    0.07635  -0.597    0.551
Condition_C1         -0.06480    0.09550  -0.678    0.499
Condition_C2          0.04562    0.05281   0.864    0.389
Tiringc               0.02525    0.04763   0.530    0.597
Condition_C1:Tiringc -0.02932    0.05811  -0.505    0.615
Condition_C2:Tiringc -0.03945    0.03380  -1.167    0.245

Residual standard error: 0.9304 on 151 degrees of freedom
  (4 observations deleted due to missingness)
Multiple R-squared:  0.02343,	Adjusted R-squared:  -0.008906 
F-statistic: 0.7246 on 5 and 151 DF,  p-value: 0.606
```

```
Call:
lm(formula = FFFSZ ~ Condition_C1 + Condition_C2 + Tiringc +
    Condition_C1:Tiringc + Condition_C2:Tiringc, data = RSTPQ)

Residuals:
    Min      1Q  Median      3Q     Max
-1.7514 -0.6786 -0.1266  0.6610  2.6951

Coefficients:
                     Estimate Std. Error t value Pr(>|t|)
(Intercept)          -0.02865    0.07462  -0.384    0.702
Condition_C1         -0.04254    0.09334  -0.456    0.649
Condition_C2         -0.04612    0.05161  -0.894    0.373
Tiringc               0.07615    0.04654   1.636    0.104
Condition_C1:Tiringc  0.08747    0.05679   1.540    0.126
Condition_C2:Tiringc  0.02098    0.03303   0.635    0.526

Residual standard error: 0.9093 on 151 degrees of freedom
  (4 observations deleted due to missingness)
Multiple R-squared:  0.0411,	Adjusted R-squared:  0.009345
F-statistic: 1.294 on 5 and 151 DF,  p-value: 0.2693
```

2 Study 2

2.1 Evaluations

```
Linear mixed model fit by REML
t-tests use  Satterthwaite approximations to degrees of freedom ['lmerMod']
Formula: EvalZ ~ Condition_C1 + Condition_C2 + (1 | pp) + (1 | Perso)
   Data: EVAL_Mn

REML criterion at convergence: 12771.6

Scaled residuals:
    Min      1Q  Median      3Q     Max
-3.6941 -0.6461  0.0313  0.6449  4.1470

Random effects:
 Groups   Name        Variance Std.Dev.
 pp       (Intercept) 0.2128   0.4613
 Perso    (Intercept) 0.2724   0.5219
 Residual             0.5267   0.7257
Number of obs: 5529, groups:  pp, 189; Perso, 30

Fixed effects:
               Estimate Std. Error         df t value Pr(>|t|)
(Intercept)    0.002529   0.101503  36.470000   0.025    0.980
Condition_C1  -0.043276   0.042536 186.430000  -1.017    0.310
Condition_C2   0.002444   0.024913 185.660000   0.098    0.922
```

2.2 Action tendencies.

```
Linear mixed model fit by REML
t-tests use  Satterthwaite approximations to degrees of freedom ['lmerMod']
Formula: VAAST.RT.logZ ~ Condition_C1 * Condition_C2 * Movementc + (1 |      pp) + (1 | First_Name)
   Data: VAAST_Mn

REML criterion at convergence: 43272

Scaled residuals:
    Min      1Q  Median      3Q     Max
-4.2732 -0.6512 -0.1264  0.5113  4.7314

Random effects:
 Groups     Name        Variance Std.Dev.
 pp         (Intercept) 0.30775  0.5548
 First_Name (Intercept) 0.01315  0.1147
 Residual               0.68513  0.8277
Number of obs: 17268, groups:  pp, 189; First_Name, 24

Fixed effects:
                         Estimate Std. Error        df t value Pr(>|t|)
(Intercept)             9.246e-03  4.711e-02  1.640e+02   0.196    0.845
Condition_C1            2.049e-02  4.968e-02  1.860e+02   0.412    0.681
Condition_C2            3.145e-02  2.913e-02  1.860e+02   1.080    0.282
Movementc              -1.218e-01  1.262e-02  1.705e+04  -9.656   <2e-16 ***
Condition_C1:Movementc -7.258e-04  1.534e-02  1.705e+04  -0.047    0.962
Condition_C2:Movementc -1.472e-02  8.986e-03  1.705e+04  -1.638    0.101
---
Signif. codes:  0 '***' 0.001 '**' 0.01 '*' 0.05 '.' 0.1 ' ' 1
```

2.3 Neuropsychological systems.

```
Call:
lm(formula = BASZ ~ Condition_C1 + Condition_C2, data = RSTPQ)

Residuals:
     Min       1Q   Median       3Q      Max
-3.15586 -0.59695  0.02167  0.76199  2.07854

Coefficients:
             Estimate Std. Error t value Pr(>|t|)
(Intercept)   0.04594    0.07303   0.629    0.530
Condition_C1  0.03350    0.08849   0.379    0.705
Condition_C2  0.02558    0.05219   0.490    0.625

Residual standard error: 0.9921 on 182 degrees of freedom
Multiple R-squared:  0.00205,   Adjusted R-squared:  -0.008916
F-statistic: 0.187 on 2 and 182 DF,  p-value: 0.8296
```

```
Call:
lm(formula = FFFSZ ~ Condition_C1 + Condition_C2, data = RSTPQ)

Residuals:
     Min       1Q   Median       3Q      Max
-2.02662 -0.82462  0.03395  0.74219  2.43796

Coefficients:
              Estimate Std. Error t value Pr(>|t|)
(Intercept)   0.028762   0.074068   0.388    0.698
Condition_C1 -0.004320   0.089741  -0.048    0.962
Condition_C2  0.008565   0.052929   0.162    0.872

Residual standard error: 1.006 on 182 degrees of freedom
Multiple R-squared:  0.000159,  Adjusted R-squared:  -0.01083
F-statistic: 0.01447 on 2 and 182 DF,  p-value: 0.9856
```

3 Complementary analyses.

3.1 Complementary Analyses of Study 1

3.1.1 Evaluations.

```
Linear mixed model fit by REML
t-tests use  Satterthwaite approximations to degrees of freedom ['lmerMod']
Formula: EvalZ ~ Condition_C1 * Condition_C2 * Presencec * Tiringc + (1 |      pp) + (1 | Perso)
   Data: EVAL_Mn_Psce

REML criterion at convergence: 10599.7

Scaled residuals: 
    Min      1Q  Median      3Q     Max 
-3.5067 -0.6351  0.0128  0.6740  3.9410 

Random effects:
 Groups   Name        Variance Std.Dev.
 pp       (Intercept) 0.1597   0.3996  
 Perso    (Intercept) 0.2686   0.5183  
 Residual             0.5689   0.7542  
Number of obs: 4420, groups:  pp, 157; Perso, 30

Fixed effects:
                                 Estimate Std. Error         df t value Pr(>|t|)  
(Intercept)                    -2.999e-02  1.013e-01  3.686e+01  -0.296   0.7689  
Condition_C1                   -2.059e-02  4.485e-02  1.461e+02  -0.459   0.6469  
Condition_C2                   -2.078e-02  2.523e-02  1.442e+02  -0.824   0.4116  
Presencec                       7.208e-03  2.841e-03  1.455e+02   2.537   0.0122 *
Tiringc                         7.132e-03  2.260e-02  1.450e+02   0.316   0.7528  
Condition_C1:Presencec         -7.129e-04  3.680e-03  1.463e+02  -0.194   0.8466  
Condition_C2:Presencec          1.352e-03  1.886e-03  1.445e+02   0.717   0.4748  
Condition_C1:Tiringc           -1.988e-02  2.766e-02  1.460e+02  -0.718   0.4736  
Condition_C2:Tiringc           -1.608e-03  1.599e-02  1.440e+02  -0.101   0.9200  
Presencec:Tiringc               1.899e-03  1.968e-03  1.456e+02   0.965   0.3362  
Condition_C1:Presencec:Tiringc  5.611e-03  2.522e-03  1.465e+02   2.225   0.0276 *
Condition_C2:Presencec:Tiringc -3.276e-03  1.324e-03  1.445e+02  -2.475   0.0145 *
---
Signif. codes:  0 '***' 0.001 '**' 0.01 '*' 0.05 '.' 0.1 ' ' 1
```

3.1.2 Action tendencies.

```
Linear mixed model fit by REML
t-tests use  Satterthwaite approximations to degrees of freedom ['lmerMod']
Formula: VAAST.RT.logZ ~ Condition_C1 * Condition_C2 * Movementc * Tiringc *
    Presencec + (1 | pp) + (1 | First_Name)
   Data: VAAST_Mn_Psce

REML criterion at convergence: 36370.5

Scaled residuals:
    Min      1Q  Median      3Q     Max
-4.1839 -0.6627 -0.1253  0.5356  4.4435

Random effects:
 Groups     Name        Variance Std.Dev.
 pp         (Intercept) 0.28201  0.5310
 First_Name (Intercept) 0.01294  0.1138
 Residual               0.68536  0.8279
Number of obs: 14453, groups:  pp, 157; First_Name, 24

Fixed effects:
                                            Estimate Std. Error         df t value Pr(>|t|)
(Intercept)                               -5.350e-02  5.138e-02  1.530e+02  -1.041  0.29937
Condition_C1                              -5.716e-02  5.676e-02  1.450e+02  -1.007  0.31557
Condition_C2                               8.663e-03  3.204e-02  1.450e+02   0.270  0.78726
Movementc                                 -1.245e-01  1.468e-02  1.426e+04  -8.483  < 2e-16 ***
Tiringc                                    5.591e-02  2.865e-02  1.450e+02   1.951  0.05297 .
Presencec                                  4.558e-03  3.599e-03  1.450e+02   1.266  0.20742
Condition_C1:Movementc                     1.745e-02  1.820e-02  1.426e+04   0.959  0.33756
Condition_C2:Movementc                    -1.089e-03  1.025e-02  1.426e+04  -0.106  0.91535
Condition_C1:Tiringc                      -1.299e-02  3.502e-02  1.450e+02  -0.371  0.71120
Condition_C2:Tiringc                      -3.251e-03  2.031e-02  1.450e+02  -0.160  0.87302
Movementc:Tiringc                         -8.692e-03  9.187e-03  1.426e+04  -0.946  0.34412
Condition_C1:Presencec                    -2.454e-03  4.656e-03  1.450e+02  -0.527  0.59895
Condition_C2:Presencec                     3.959e-03  2.394e-03  1.450e+02   1.654  0.10029
Movementc:Presencec                        3.737e-03  1.154e-03  1.426e+04   3.237  0.00121 **
Tiringc:Presencec                         -4.903e-03  2.493e-03  1.450e+02  -1.967  0.05111 .
Condition_C1:Movementc:Tiringc            -8.806e-03  1.125e-02  1.426e+04  -0.783  0.43362
Condition_C2:Movementc:Tiringc            -1.190e-02  6.499e-03  1.426e+04  -1.831  0.06710 .
Condition_C1:Movementc:Presencec           1.291e-03  1.494e-03  1.426e+04   0.865  0.38726
Condition_C2:Movementc:Presencec          -2.146e-03  7.675e-04  1.426e+04  -2.796  0.00517 **
Condition_C1:Tiringc:Presencec            -1.912e-03  3.189e-03  1.450e+02  -0.600  0.54969
Condition_C2:Tiringc:Presencec            -1.457e-03  1.680e-03  1.450e+02  -0.867  0.38717
Movementc:Tiringc:Presencec               -2.146e-03  8.018e-04  1.426e+04  -2.676  0.00745 **
Condition_C1:Movementc:Tiringc:Presencec   2.559e-03  1.027e-03  1.426e+04   2.493  0.01269 *
Condition_C2:Movementc:Tiringc:Presencec  -6.360e-04  5.399e-04  1.426e+04  -1.178  0.23882
---
Signif. codes:  0 ‘***’ 0.001 ‘**’ 0.01 ‘*’ 0.05 ‘.’ 0.1 ‘ ’ 1
```

3.1.3 Neuropsychological systems.

```
Call:
lm(formula = BASZ ~ Condition_C1 * Condition_C2 * Tiringc * Presencec,
    data = RSTPQ_Psce)

Residuals:
     Min       1Q   Median       3Q      Max
-2.23219 -0.55353  0.08336  0.56758  2.08705

Coefficients: (4 not defined because of singularities)
                                      Estimate Std. Error t value Pr(>|t|)
(Intercept)                         -0.1114125  0.0751670  -1.482  0.14047
Condition_C1                        -0.0174565  0.0931570  -0.187  0.85162
Condition_C2                         0.0268642  0.0525104   0.512  0.60972
Tiringc                              0.0728175  0.0470601   1.547  0.12398
Presencec                            0.0222855  0.0058966   3.779  0.00023 ***
Condition_C1:Tiringc                -0.0363832  0.0575948  -0.632  0.52858
Condition_C2:Tiringc                -0.0374926  0.0333006  -1.126  0.26209
Condition_C1:Presencec               0.0045863  0.0076285   0.601  0.54865
Condition_C2:Presencec              -0.0019117  0.0039207  -0.488  0.62659
Tiringc:Presencec                   -0.0075752  0.0040827  -1.855  0.06558 .
Condition_C1:Tiringc:Presencec       0.0092785  0.0052244   1.776  0.07785 .
Condition_C2:Tiringc:Presencec       0.0007923  0.0027514   0.288  0.77379
---
Signif. codes:  0 '***' 0.001 '**' 0.01 '*' 0.05 '.' 0.1 ' ' 1

Residual standard error: 0.8809 on 144 degrees of freedom
  (4 observations deleted due to missingness)
Multiple R-squared:  0.1549,	Adjusted R-squared:  0.09029
F-statistic: 2.399 on 11 and 144 DF,  p-value: 0.009216
```

```
Call:
lm(formula = FFFSZ ~ Condition_C1 * Condition_C2 * Tiringc *
    Presencec, data = RSTPQ_Psce)

Residuals:
    Min      1Q  Median      3Q     Max
-1.8156 -0.6498 -0.1314  0.5968  2.6638

Coefficients: (4 not defined because of singularities)
                                      Estimate Std. Error t value Pr(>|t|)
(Intercept)                          0.0183080  0.0770027   0.238   0.8124
Condition_C1                        -0.0888746  0.0954320  -0.931   0.3533
Condition_C2                        -0.0459282  0.0537928  -0.854   0.3946
Tiringc                              0.0646891  0.0482094   1.342   0.1818
Presencec                           -0.0070582  0.0060406  -1.168   0.2446
Condition_C1:Tiringc                 0.1246942  0.0590014   2.113   0.0363 *
Condition_C2:Tiringc                 0.0162347  0.0341139   0.476   0.6349
Condition_C1:Presencec               0.0082180  0.0078148   1.052   0.2948
Condition_C2:Presencec              -0.0000895  0.0040165  -0.022   0.9823
Tiringc:Presencec                    0.0092656  0.0041824   2.215   0.0283 *
Condition_C1:Tiringc:Presencec      -0.0049058  0.0053520  -0.917   0.3609
Condition_C2:Tiringc:Presencec      -0.0029267  0.0028186  -1.038   0.3008
---
Signif. codes:  0 '***' 0.001 '**' 0.01 '*' 0.05 '.' 0.1 ' ' 1

Residual standard error: 0.9025 on 144 degrees of freedom
  (4 observations deleted due to missingness)
Multiple R-squared:  0.09676,	Adjusted R-squared:  0.02776
F-statistic: 1.402 on 11 and 144 DF,  p-value: 0.1778
```

3.2 Complementary Analyses of Study 2

3.2.1 Evaluations.

```
Linear mixed model fit by REML
t-tests use  Satterthwaite approximations to degrees of freedom ['lmerMod']
Formula: EvalZ ~ Condition_C1 * Condition_C2 * Presencec + (1 | pp) +      (1 | Perso)
   Data: EVAL_Mn

REML criterion at convergence: 12678.6

Scaled residuals: 
    Min      1Q  Median      3Q     Max 
-3.6807 -0.6462  0.0277  0.6410  4.1649 

Random effects:
 Groups   Name        Variance Std.Dev.
 pp       (Intercept) 0.1959   0.4426  
 Perso    (Intercept) 0.2744   0.5238  
 Residual             0.5297   0.7278  
Number of obs: 5469, groups:  pp, 187; Perso, 30

Fixed effects:
                         Estimate Std. Error         df t value Pr(>|t|)   
(Intercept)            -6.823e-03  1.016e-01  3.607e+01  -0.067  0.94682   
Condition_C1           -6.053e-02  4.175e-02  1.814e+02  -1.450  0.14884   
Condition_C2            1.381e-03  2.438e-02  1.806e+02   0.057  0.95487   
Presencec               7.320e-03  2.429e-03  1.807e+02   3.013  0.00296 **
Condition_C1:Presencec  1.339e-03  3.007e-03  1.809e+02   0.445  0.65675   
Condition_C2:Presencec  1.476e-04  1.699e-03  1.804e+02   0.087  0.93090   
---
Signif. codes:  0 ‘***’ 0.001 ‘**’ 0.01 ‘*’ 0.05 ‘.’ 0.1 ‘ ’ 1
```

3.2.2 Action tendencies.

```
Linear mixed model fit by REML
t-tests use  Satterthwaite approximations to degrees of freedom ['lmerMod']
Formula: VAAST.RT.logZ ~ Condition_C1 * Condition_C2 * Movementc * Presencec +      (1 | pp) + (1 | First_Name)
   Data: VAAST_Mn_RV

REML criterion at convergence: 42858.9

Scaled residuals: 
    Min      1Q  Median      3Q     Max 
-4.2764 -0.6518 -0.1260  0.5118  4.7230 

Random effects:
 Groups     Name        Variance Std.Dev.
 pp         (Intercept) 0.2964   0.5445  
 First_Name (Intercept) 0.0132   0.1149  
 Residual               0.6850   0.8276  
Number of obs: 17083, groups:  pp, 187; First_Name, 24

Fixed effects:
                                   Estimate Std. Error         df t value Pr(>|t|)    
(Intercept)                       1.208e-02  4.710e-02  1.610e+02   0.256  0.79794    
Condition_C1                      4.141e-02  4.972e-02  1.810e+02   0.833  0.40602    
Condition_C2                      3.107e-02  2.906e-02  1.810e+02   1.069  0.28647    
Movementc                        -1.178e-01  1.284e-02  1.687e+04  -9.172  < 2e-16 ***
Presencec                        -7.764e-03  2.896e-03  1.810e+02  -2.681  0.00803 ** 
Condition_C1:Movementc            3.753e-03  1.564e-02  1.687e+04   0.240  0.81036    
Condition_C2:Movementc           -1.422e-02  9.134e-03  1.687e+04  -1.557  0.11948    
Condition_C1:Presencec            1.904e-03  3.585e-03  1.810e+02   0.531  0.59603    
Condition_C2:Presencec            2.194e-03  2.026e-03  1.810e+02   1.083  0.28036    
Movementc:Presencec              -1.091e-05  9.122e-04  1.687e+04  -0.012  0.99045    
Condition_C1:Movementc:Presencec -1.552e-03  1.131e-03  1.687e+04  -1.373  0.16988    
Condition_C2:Movementc:Presencec  1.037e-03  6.369e-04  1.687e+04   1.628  0.10361    
---
Signif. codes:  0 ‘***’ 0.001 ‘**’ 0.01 ‘*’ 0.05 ‘.’ 0.1 ‘ ’ 1
```

3.2.3 Neuropsychological systems.

```
Call:
lm(formula = BASZ ~ Condition_C1 + Condition_C2 + Presencec +
    Condition_C1:Presencec + Condition_C2:Presencec, data = RSTPQ)

Residuals:
     Min       1Q   Median       3Q      Max
-3.00908 -0.61248  0.08433  0.68432  2.03997

Coefficients:
                         Estimate Std. Error t value Pr(>|t|)
(Intercept)             0.0238180  0.0717528   0.332 0.740319
Condition_C1           -0.0137195  0.0872793  -0.157 0.875272
Condition_C2            0.0481685  0.0510807   0.943 0.346958
Presencec               0.0176099  0.0050951   3.456 0.000684 ***
Condition_C1:Presencec  0.0124599  0.0062708   1.987 0.048452 *
Condition_C2:Presencec  0.0001602  0.0035850   0.045 0.964413
---
Signif. codes:  0 ‘***’ 0.001 ‘**’ 0.01 ‘*’ 0.05 ‘.’ 0.1 ‘ ’ 1

Residual standard error: 0.9636 on 179 degrees of freedom
Multiple R-squared:  0.07408,   Adjusted R-squared:  0.04821
F-statistic: 2.864 on 5 and 179 DF,  p-value: 0.01632
```

```
Call:
lm(formula = FFFSZ ~ Condition_C1 + Condition_C2 + Presencec +
    Condition_C1:Presencec + Condition_C2:Presencec, data = RSTPQ)

Residuals:
     Min       1Q   Median       3Q      Max
-2.02166 -0.81797 -0.01198  0.78909  2.38298

Coefficients:
                        Estimate Std. Error t value Pr(>|t|)
(Intercept)             0.040675   0.075139   0.541    0.589
Condition_C1            0.005867   0.091398   0.064    0.949
Condition_C2            0.006210   0.053491   0.116    0.908
Presencec               0.001907   0.005336   0.357    0.721
Condition_C1:Presencec -0.008016   0.006567  -1.221    0.224
Condition_C2:Presencec  0.001848   0.003754   0.492    0.623

Residual standard error: 1.009 on 179 degrees of freedom
Multiple R-squared:  0.01092,   Adjusted R-squared:  -0.01671
F-statistic: 0.3953 on 5 and 179 DF,  p-value: 0.8516
```
